# Dissecting the Distinct Tumor Microenvironments of HRD and HRP Ovarian Cancer: Implications for Targeted Therapies to Overcome PARPi Resistance in HRD Tumors and Refractoriness in HRP Tumors

**DOI:** 10.1002/advs.202309755

**Published:** 2024-08-13

**Authors:** Junjun Qiu, Tingting Ren, Qinqin Liu, Qian Jiang, Tong Wu, Leong Chi Cheng, Wenqing Yan, Xinyu Qu, Xiao Han, Keqin Hua

**Affiliations:** ^1^ Department of Gynecology Obstetrics and Gynecology Hospital Fudan University 419 Fangxie Road Shanghai 200011 China; ^2^ Shanghai Key Laboratory of Female Reproductive Endocrine‐Related Diseases 413 Zhaozhou Road Shanghai 200011 China; ^3^ Kangxiang Bio‐tech.Ltd. 2168 Chenhang Road ShangHai 201114 China

**Keywords:** high grade tubo‐ovarian cancer, HRD, HRP, precise treatment strategies, single‐cell RNA sequencing, single‐cell T cell receptor sequencing, tumor immune microenvironment

## Abstract

High‐grade serous tubo‐ovarian cancer (HGSTOC) is an aggressive gynecological malignancy including homologous recombination deficient (HRD) and homologous recombination proficient (HRP) groups. Despite the therapeutic potential of poly (ADP‐ribose) polymerase inhibitors (PARPis) and anti‐PDCD1 antibodies, acquired resistance in HRD and suboptimal response in HRP patients necessitate more precise treatment. Herein, single‐cell RNA and single‐cell T‐cell receptor sequencing on 5 HRD and 3 HRP tumors are performed to decipher the heterogeneous tumor immune microenvironment (TIME), along with multiplex immunohistochemistry staining and animal experiments for validation. HRD tumors are enriched with immunogenic epithelial cells, FGFR1+PDGFRβ+ myCAFs, M1 macrophages, tumor reactive CD8+/CD4+ Tregs, whereas HRP tumors are enriched with HDAC1‐expressing epithelial cells, indolent CAFs, M2 macrophages, and bystander CD4+/CD8+ T cells. Significantly, customized therapies are proposed. For HRD patients, targeting FGFR1+PDGFRβ+ myCAFs via tyrosine kinase inhibitors, targeting Tregs via anti‐CCR8 antibodies/TNFRSF4 stimulation, and targeting CXCL13+ exhausted T cells by blocking PDCD1/CTLA‐4/LAG‐3/TIGIT are proposed. For HRP patients, targeting indolent CAFs, targeting M2 macrophages via CSF‐1/CSF‐1R inhibitors, targeting bystander T cells via tumor vaccines, and targeting epithelial cells via HDAC inhibitors. The study provides comprehensive insights into HRD and HRP TIME and tailored therapeutic approaches, addressing the challenges of PARPi‐resistant HRD and refractory HRP tumors.

## Graphical abstract

A comprehensive single‐cell atlas of homologous recombination deficient (HRD) and homologous recombination proficient (HRP) ovarian cancer is depicted. Through verification of mIHC and in‐vivo xenograft validation, the distinct tumor immune microenvironment is unveiled, the promising targets specifically for HRD and HRP tumors are proposed, providing novel insights for developing effective clinical therapies customized for HRD and HRP patients.

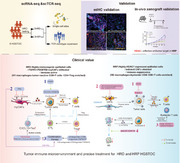

## 1 Introduction

High‐grade serous tubo‐ovarian carcinoma (HGSTOC) represents one of the most aggressive gynecological malignancies, with over 300000 estimated new cases and 190000 disease‐specific deaths reported worldwide in 2020.^[^ ^1^ ^]^ Most HGSTOC patients are diagnosed at an advanced stage, and the five‐year overall survival is only approximately 30%.^[^ ^2^ ^]^ Currently, the primary treatment for HGSTOC is cytoreductive surgery combined with platinum‐based chemotherapy.^[^ ^3^ ^]^ Unfortunately, over 75% of patients relapse within two years due to the high risk of resistance to chemotherapy.^[^ ^3^, ^4^ ^]^ Therefore, there is an urgent need to explore more effective therapies for HGSTOC.

According to the deficiency status in the homologous recombination DNA repair pathway, HGSTOC patients can be stratified into homologous recombination deficient (HRD) and homologous recombination proficient (HRP) subgroups.^[^ ^5^ ^]^ Poly (ADP‐ribose) polymerase inhibitors (PARPis) have substantially improved the progression‐free survival rates of HRD patients in clinical trials and are recommended as maintenance therapy for those who achieved complete response or partial response after platinum‐based chemotherapy.^[^ ^6^ ^]^ However, the emergence of acquired resistance in a fraction of patients limited the efficacy of PARPi.^[^ ^7^ ^]^ As a result of both the limited efficacy of PARPis owing to insufficient synthetic lethality and the lack of effective therapeutic regimens, there is an urgent need for novel clinical treatment strategies for HRP patients.^[^ ^8^, ^9^ ^]^ Therefore, it is essential to identify novel targets unique to each subtype and explore more effective targeted therapies customized to HRD and HRP patients to improve their prognosis.

With the advent of cancer immunotherapy, immune checkpoint blockade (ICB), especially anti‐PDCD1 therapy, has been explored as a treatment option for PARPi‐resistant or HRP HGSTOC patients; however, their therapeutic efficacy has been limited thus far.^[^ ^10^ ^]^ In HRD patients, PARPis combined with anti‐PDCD1 therapy showed limited improvement compared to PARPi monotherapy.^[^ ^11^, ^12^ ^]^ In HRP patients, a PARPi+anti‐PDCD1 combination treatment exhibited a higher objective response rate (ORR: 27%) compared to those of anti‐PDCD1 monotherapy (ORR: 10%) and PARPi monotherapy (ORR: 5%),^[^ ^11^, ^12^, ^13^ ^]^ but this combination regimen did not improve overall survival.^[^ ^12^, ^13^ ^]^ Previous studies also proved that these unsatisfactory tumor responses to combination therapies are likely attributable to tumor immune microenvironment (TIME) heterogeneity, which has not been fully elucidated in HRD and HRP HGSTOC patients.^[^ ^14^ ^]^ Hence, to address this clinical challenge, it is imperative to decipher the heterogeneity of the TIME and molecular intricacies within discrete HRD and HRP subsets and further explore effective and precise therapeutic strategies.

Currently, single‐cell RNA sequencing (scRNA‐seq) has provided unprecedented insights into the cellular composition and intratumor heterogeneity within the TIME.^[^ ^15^ ^]^ Moreover, single‐cell T‐cell receptor sequencing (scTCR‐seq) can further deepen the understanding of the immune landscape by providing information on T‐cell clonality.^[^ ^16^ ^]^ Although several scRNA‐seq studies have revealed ovarian cancer heterogeneity and identified several novel markers for predicting prognosis in ovarian cancer,^[^ ^17^, ^18^ ^]^ coupled scRNA‐seq and scTCR‐seq analyses to further depict TIME heterogeneity in HRD and HRP patients and guide their respective potential precise therapies have yet to be performed.

Herein, for the first time, we conducted integrated scRNA‐seq and scTCR‐seq analyses of HRD and HRP HGSTOC tumors and found that HRD tumors were highly enriched in immunogenic epithelial cells, infiltrated FGFR1+PDGFRβ+ myCAFs, M1 macrophages, tumor reactive CD8+ T cells, and CD4+ Tregs, while HRP tumors were highly enriched in HDAC1‐expressing epithelial cells, indolent CAFs, M2 macrophages, and bystander CD4+/CD8+ T cells. Additionally, we proposed potential therapies customized to HRD and HRP patients targeting epithelial cells, fibroblasts and immune cells based on their divergent tumor microenvironment profiles. Specifically, for HRD HGSTOC, in order to break through the treatment dilemma of PARPi resistance and limited improvement in clinical outcomes with the combination of PARPi and anti‐PDCD1, we proposed administering combination therapy targeting Tregs/FGFR1+ PDGFRβ+ myCAFs and reactivating exhausted CD8+ T cells (Tex cells). For HRP HGSTOC, in order to explore effective therapies, we not only proposed that HDAC inhibitors (HDACis) may serve as an effective therapeutic strategy, which was validated by animal experiments, but also innovatively discovered the existence of a physical barrier formed by “indolent CAFs” surrounding tumor cells, confirmed by multiplex immunohistochemistry (mIHC), which might serve as a promising target. All these findings not only illuminated the unique TIME of HRD and HRP tumors but also proposed precise targeted therapies, aside from PARPis and anti‐PDCD1 antibodies, for HRD and HRP patients respectively, laying the framework for future clinical trials.

## 2 Results

### 2.1 Single‐Cell Transcriptome Atlas of HRD and HRP HGSTOC

**Figure 1:**
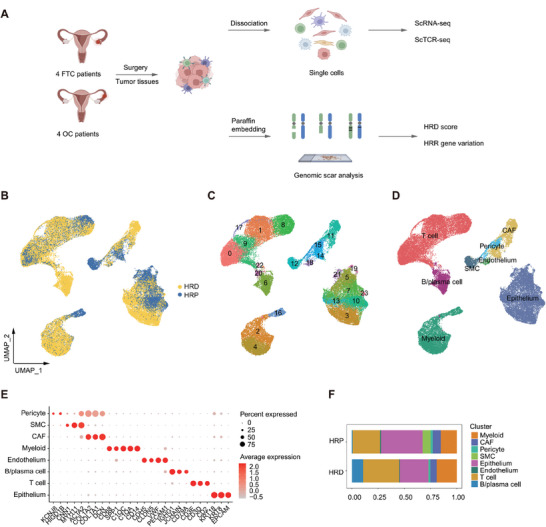
Single‐Cell transcriptome atlas of HRD and HRP HGSTOC. A) A schematic diagram illustrating the process of collecting and analyzing single‐cell profiles and genomic scars from eight HGSTOC biopsies. B) A UMAP plot demonstrating 45159 cells from 8 HGSTOC patient origins, colored by HRD status of each patient. C) A UMAP plot demonstrating 45159 cells grouped into 24 clusters. D) A UMAP plot demonstrating 45159 cells grouped into 8 cell types. E) A dot plot demonstrating the expression of specific marker genes of each cell type. Dot size represented the percentage of marker gene expressed cells. Dot color represented the average expression level of marker genes. Red, higher expression; grey, lower expression. F) A column plot demonstrating the average proportion of different cell types in HRD and HRP HGSTOC samples.

As the overall workflow shows (**Figure** **1**A), we collected tumor tissues from eight HGSTOC patients, including four ovarian cancer (OC) and four fallopian tube cancer (FTC) patients. After evaluating HRD status and BRCA1/2 gene mutations using genomic scar analysis (GSA) technology, we categorized these samples into two groups (Figure 1A,B): the HRD group (BRCAmu OC3/FTC2 and BRCAwt OC2/FTC3/FTC4) and the HRP group (BRCAwt OC1/OC4/FTC1). To investigate the TIME and cellular heterogeneity of the two groups, scRNA‐seq and paired scTCR‐seq were performed (Figure 1A). Following quality control assessment, a total of 45159 cells were retained and grouped into 24 clusters (Figure 1C).

We identified eight major cell types (Figure 1D) based on the expression of cell‐type marker genes (Figure 1E), including epithelial cells (EPCAM, KRT18 and KRT8), CAFs (DCN, COL1A1 and COL1A2), T cells (CD2, CD3D, and CD3E), B/plasma cells (CD79A, IGHG1 and JCHAIN), endothelial cells (PECAM1, VWF, CLDN5 and CDH5), myeloid cells (CD14, C1QA, C1QC, SPP1 and CD68), smooth muscle cells (SMCs) (ACTA2, MYH11 and CNN1) and pericytes (HIGD1B and KCNJ8). Of note, compared to HRP tumors, HRD tumors contained more immune cells, including myeloid cells, T cells and B cells (Figure 1F), which suggested that HRD patients might have an immune‐infiltrated tumor microenvironment (TME).

Subsequently, based on the cell type infiltration profiles of HRD and HRP tumors, we next focused on the main structural cells (epithelial cells, fibroblasts) and immune cells (myeloid cells, T cells) to decipher the TIME heterogeneity between the two groups in depth and explore novel precise treatments for each subgroup.

HGSTOC, High grade serous tubo‐ovarian carcinoma; HRP, Homologous recombination proficiency; HRD, Homologous recombination deficiency; OC, Ovarian cancer; FTC, Fallopian tube cancer; CAF, Cancer‐associated fibroblasts; SMC, Smooth muscle cells; UMAP, Uniform manifold approximation and projection; scRNA‐seq, Single‐cell RNA sequencing; scTCR‐seq, Single‐cell TCR sequencing.

### 2.2 Epithelial Cells with Great Stemness in the HRD Group Versus Epithelial Cells with High HDAC1 Expression in the HRP Group

**Figure 2:**
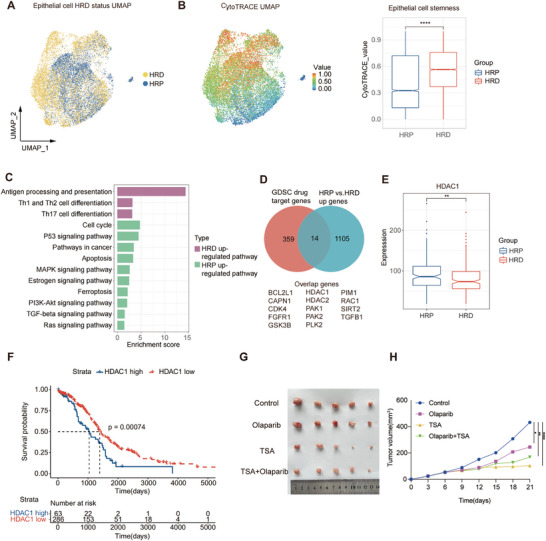
Epithelial cells: epithelial cells with great stemness in HRD group versus epithelial cells highly expressing HDAC1 in HRP group. A) A UMAP plot demonstrating epithelial cells from eight HGSTOC patient origins, colored by HRD status. Yellow, HRD; blue, HRP. B) CytoTRACE value UMAP and boxplot demonstrating distinct stemness of epithelial cells from HRD and HRP groups. The color gradient indicates the cytotrace stemness value (red, higher stemness; blue, lower stemness). P value was calculated by a Wilcoxon signed rank test. ****P < 0.0001. C) A bar plot demonstrating the enriched pathways of upregulated gene expression in HRD and HRP epithelial cells, respectively. Purple, HRD; Green, HRP. D) A venn plot demonstrating the intersection of upregulated genes in HRP epithelial cells versus HRD epithelial cells and drug target genes from GDSC database. E) A box plot demonstrating the higher expression of HDAC1 in HRP tumors compared to HRD tumors in TCGA bulk RNA seq datasets. P value was calculated by a Wilcoxon signed rank test. **P < 0.01. F) Kaplan–Meier overall survival curves illustrating the prognostic value of HDAC1 gene expression, validated in TCGA HGSTOC cohorts. P value was calculated by a log‐rank test. G) Photographs of tumor sizes attained from SKOV3 xenograft nude mices by indicated treatment (*n* = 5 for each group). H) A growth curve demonstrating superior tumor control effects of the HDAC inhibitor (TSA) and the combination therapy of PARPis + TSA in comparison to the control group (*n* = 5 for each group). P value was calculated by two‐way ANOVA analysis with Tukey's pairwise comparisons. *Adjusted P < 0.05, **Adjusted P < 0.01, **Adjusted *P* < 0.0001).

To characterize the phenotypic heterogeneity of epithelial cells in HRD and HRP HGSTOC, we reclustered epithelial cells (Figure S1A, Supporting Information) and represented them by their grouping (**Figure** **2**A). Notably, HRD epithelial cells exhibited high differentiation potential, demonstrating their stemness (Figure 2B). Remarkably, consistent with the great immunogenicity caused by the unstable genome profiles of the HRD group,^[^ ^19^ ^]^ HRD tumor epithelial cells exhibited high expression of immune chemokines that recruit immune cells to mediate antitumor effects, such as CXCL8, CXCL10 and CXCL11 (Table S2, Supporting Information). Accordingly, HRD tumor epithelial cells were found to be greatly involved in the antigen presentation process and Th1, Th2, and Th17 cell differentiation (Figure 2C), further confirming the active immune response in the TIME of HRD HGSTOC. In contrast, HRP tumor epithelial cells were found to be strongly associated with cancer‐related pathways (Figure 2C), including the cell cycle, P53 signaling, MAPK signaling and PI3K‐AKT signaling pathways, among which the MAPK signaling pathway was reported to be related to tumor cell immune evasion.^[^ ^20^ ^]^ Therefore, these preliminary results suggest that the HRD group has an immune‐active phenotype and the HRP group has an immune evasion phenotype, which could partially explain their different clinical outcomes and treatment sensitivities and, more importantly, highlight the necessity of developing more precise treatments for the two HGSTOC groups, particularly for HRP tumors, as they are more resistant to PARPis.

To investigate potential new targeted therapies for HRP patients, we compared the upregulated differentially expressed genes (DEGs) identified in HRP versus HRD epithelial cells with known drug target genes in the Genomics of Drug Sensitivity in Cancer (GDSC) database.^[^ ^21^ ^]^ Eventually, 14 upregulated drug target genes were identified (Figure 2D). Among them, HDAC1, SIRT2, TGFB1, and GSK3B were significantly upregulated in HRP tumors and associated with unfavorable survival outcomes of patients according to The Cancer Genome Atlas (TCGA) database (Figure 2E,F; Figure S1B–G, Supporting Information). These findings suggested that inhibitors targeting these identified genes might be effective therapeutic strategies for HRP tumors. Among them, given that HDACis have been shown to induce DNA damage during DNA replication, leading to increased tumor neoantigen and active antitumor effects,^[^ ^22^ ^]^ we established subcutaneous and intra‐peritoneal xenograft tumor model using HRP ovarian cancer cell line SKOV3, which has high expression level of HDAC1 (Figure S1H,I, Supporting Information) and evaluated its response to different treatment modalities. Notably, based on the subcutaneous xenograft model, we found that while PARPi (olaparib) exhibited efficacy in inhibiting tumor growth compared to the control group, the HDACi Trichostatin A (TSA) monotherapy and the combination of TSA with olaparib resulted in a more pronounced reduction in tumor growth (Figure 2G,H). Furthermore, base on the intraperitoneal xenograft model, we not only discovered that the TSA monotherapy demonstrated the fewest metastatic foci relative to the other treatment groups (FigureS2A,B, Supporting Information), but also identified the higher CD86 expression of macrophage cells in TSA monotherapy group (FigureS2C–E, Supporting Information), indicating that the therapeutic efficacy of TSA may be linked to alterations in the tumor microenvironment. These results suggested that TSA may have substantial potential in inhibiting both primary tumor growth and metastasis, affirming the potential clinical application of HDACis for HRP patients.

HGSTOC, High grade serous tubo‐ovarian carcinoma; HRP, Homologous recombination proficiency; HRD, Homologous recombination deficiency; UMAP, Uniform manifold approximation and projection; GDSC, Genomics of Drug Sensitivity in Cancer; TCGA, The Cancer Genome Atlas; TSA, Trichostatin A; PARPi, PARP inhibitor; HDACi, HDAC inhibitor

### 2.3 Immune‐Active CAFs in the HRD Group Versus “Indolent” CAFs in the HRP Group

**Figure 3:**
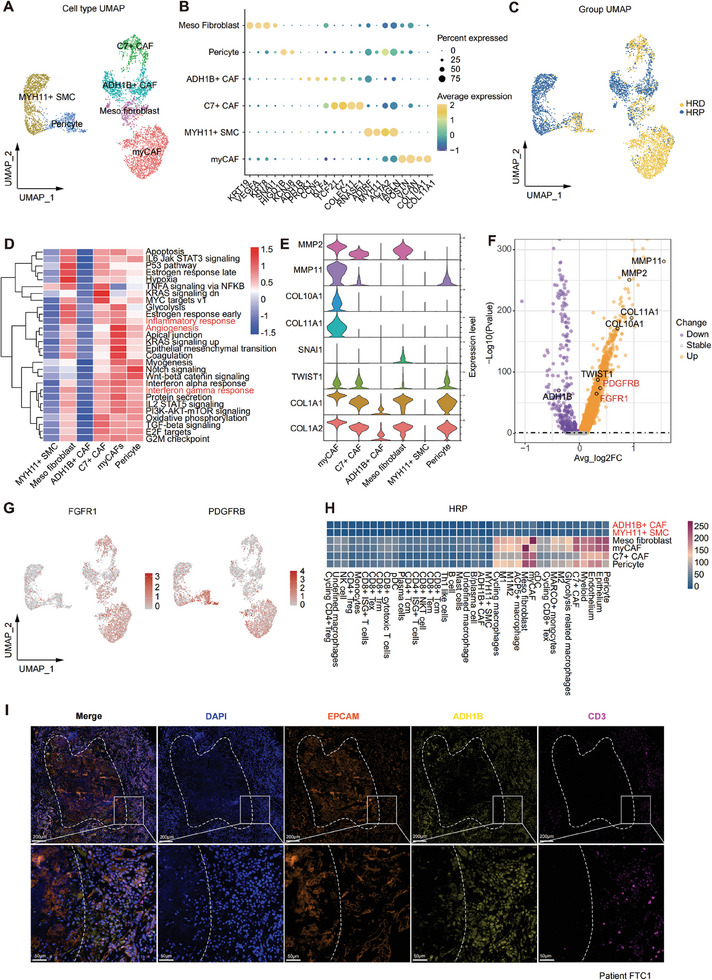
CAFs: immune‐active CAFs in HRD group versus “indolent CAFs” in HRP group. A) A UMAP plot demonstrating 4924 CAF‐SMC‐pericyte cells reclustered by 6 subtypes. B) A dot plot showing the classic marker genes of stromal cell subtypes. Dot size represented the percentage of marker gene expressed cells. Dot color represented the average expression level of marker genes (Yellow, higher expression; blue, lower expression). C) A UMAP plot representations of CAF‐SMC‐pericyte cells in HRD and HRP groups. D) A heatmap showing the enriched functional pathways of CAF‐SMC‐pericytes. Color bar represented scaled enrichment score of each subtype. Red, higher scaled enrichment score; blue, lower scaled enrichment score. E) A violin plot representing the expression signatures of collagens and MMPs that characterize different CAF‐SMC‐pericyte subtypes. F) A volcano plot demonstrating differentially expressed genes in CAFs of HRD and HRP group. The abscissa represented −log10(p value). P value was calculated by a Wilcoxon signed rank test. The ordinate represented average log2(fold change) of each cell. Purple, down regulated genes; orange, up regulated genes. G) A UMAP plot showing FGFR1 and PDGFRΒ expression levels in CAF‐SMC‐pericyte cells. Bar color represented the gene expression level (Red, higher expression; grey, lower expression). H) A cellphonedb heatmap representing the cellular interactions between CAF subtypes and other cell types (epithelial cells, myeloid cells, T cells) in HRP group. The color gradient indicates the ligand‐receptor interaction number (red, higher number; blue, lower number). I) Multiplex IHC showing the tumor epithelial cells area (EPCAM+, orange) inside the dotted line, surrounded by the indolent CAF (ADH1B+, yellow) barrier, so T cells (CD3+, mauve) are blocked by the CAF barrier and difficult to infiltrate the tumor area. Scale bar = 100 µM (upper), Scale bar = 50 µM (lower).

As the predominant stromal cell type in tumors, CAF‐SMC pericyte cells can influence immune cell infiltration and are strongly correlated with poor prognosis.^[^ ^23^ ^]^ In this study, we further reclustered CAF‐SMC pericytes and identified six subtypes (**Figure** **3**A), including MYH11+ SMCs (MYH11, ACTA2, TAGLN), pericytes (HIGD1B, KCNJ8), ADH1B+ CAFs (ADH1B, PROK1, CCN5), C7+ CAFs (C7, COLAC11, TCF21, and RNASE1), myofibroblast CAFs (myCAFs) (POSTN, VCAN, COL10A1, and COL11A1) and meso‐fibroblasts (KRT8, VEGFA, KRT19 and SNAI1).^[^ ^24^ ^]^ (Figure 3B).

Remarkably, HRD tumors exhibited high infiltration of C7+ CAFs, meso‐fibroblasts, pericytes, and myCAFs (Figure 3A,C), which actively participate in the inflammatory response, angiogenesis, and interferon α/γ response (Figure 3D). Among these subgroups, myCAFs (Figure 3E) exhibited high expression of a unique set of collagens and matrix metalloproteinases (MMPs), suggesting their potential role in remodeling the extracellular matrix (ECM) and organizing collagen fibrils.^[^ ^25^ ^]^ In addition, we noted that myCAFs in HRD tumors also featured high expression of two tyrosine kinase receptors, FGFR1 and PDGFRΒ (Figure 3F,G), which implied the potential therapeutic efficiency of tyrosine kinase inhibitors (TKIs) for HRD patients. Conversely, HRP tumors showed an abundance of ADH1B+ CAFs and MYH11+ SMCs that scarcely secreted collagens and MMPs (Figure 3E). Moreover, these cells also exhibited limited biological functions (Figure 3D) and interactions with other cell subtypes (Figure 3H). Subsequently, using mIHC staining, we explored the spatial positioning of these two cell types in HRP tumors and found that numerous ADH1B+ CAFs and MYH11+ SMCs surrounded the tumor cells, while a limited number of T cells were situated along the peripheral edge of the CAFs, forming an immune‐excluded TIME (Figure 3I, Figure S3, Supporting Information). Owing to the limited functions, scarce interactions with other cell subtypes, and spatial locations, we termed ADH1B+ CAFs and MYH11+ SMCs “indolent CAFs”, which appeared to act as a physical barrier surrounding aggregated tumor cells, preventing immune cell infiltration and obstructing further antigen presentation signals. Such results suggest that adoptive immune‐cell therapy for HRP tumors may be challenging given the strict physical barrier.

Altogether, through the in‐depth investigation of CAFs, we identified immune‐active CAFs that express two potential tyrosine kinase receptors (FGFR1 and PDGFRΒ) in the HRD group, as well as “indolent CAFs” that form an immune barrier in the HRP group, providing clinical insights for the exploration of novel therapeutic strategies targeting CAFs.

HGSTOC, High grade serous tubo‐ovarian carcinoma; HRP, Homologous recombination proficiency; HRD, Homologous recombination deficiency; CAF, Cancer‐associated fibroblasts; SMC, Smooth muscle cells; UMAP, Uniform manifold approximation and projection; FC, Fold change; MMPs, Matrix metalloproteinases; IHC, Immunohistochemistry

### 2.4 Antigen‐Presenting Macrophages in the HRD Group Versus Anti‐Inflammatory M2 Macrophages in the HRP Group

**Figure 4:**
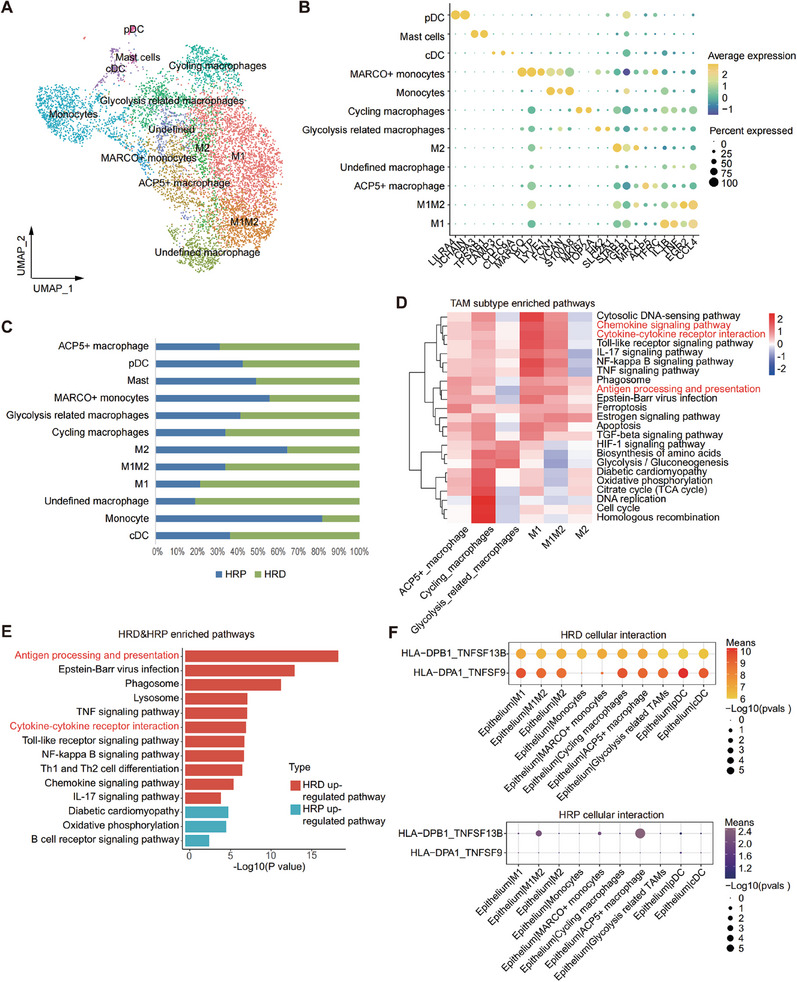
Macrophages: antigen presentation cells in HRD group versus anti‐inflammatory M2 in HRP group. A) A UMAP plot demonstrating myeloid cells reclustered into 12 subtypes. B) A dot plot showing classic marker genes of myeloid subtypes marker genes. Dot size represented the percentage of marker gene expressed cells. Dot color represented the average expression level of marker genes (Yellow, higher expression; blue, lower expression). C) A column plot showing the infiltration of 12 myeloid cell subtypes in HRD and HRP group. Blue, HRP group; green, HRD group. D) A Qusage heatmap representing enriched functional pathways of TAM subtypes with an enrichment score presented. Color bar represented with a scaled enrichment score of each subtype presented (color key from blue to red). Red, higher scaled enrichment score; blue, lower scaled enrichment score. E) A pathway barplot presenting enriched functional pathways of myeloid cells in HRD and HRP group. P value was calculated by fisher exact test. F) A cellphonedb dot plot showing receptor‐ligand interactions between epithelial cell and TAMs subtypes in HRD (upper) and HRP (lower) group. Dot size represented the p value of ligand‐receptor interaction. Dot color represented the means of ligand‐receptor interaction (Red, higher mean expression; purple, lower mean expression).

Myeloid cells, including tumor‐associated macrophages (TAMs), monocytes, dendritic cells and mast cells, are key components of the TIME. To better understand the unique characteristics of HRD and HRP HGSTOC with respect to myeloid cells, we reclustered myeloid cells and identified 12 distinct subsets based on classic markers: monocytes (FCN1, VCAN, S100A8), M1 macrophages (IL1B, TNF, EGR2), M2 macrophages (STAB1, TRFB1),^[^ ^26^, ^27^ ^]^ M1M2 macrophages (TNF, EGR2, STAB1), ACP5+ macrophages (ACP5), cycling macrophages (MKI67, TOP2A), glycolysis‐related macrophages (HK2, SLC2A1),^[^ ^28^ ^]^ MARCO+ macrophages (MARCO, PLTP, LYVE1), undefined macrophages, conventional dendritic cells (cDCs) (LAMP3, CD1C, CLEC9A), plasmacytoid dendritic cells (pDCs) (LILRA4, JCHAIN), and mast cells (CPA3, TPSAB1).^[^ ^29^ ^]^ (**Figure** **4**A,B). Among them, TAMs are capable of influencing tumor progression and constitute a large and highly adaptable population of immune cells in the TIME,^[^ ^30^ ^]^ attracting our interest.

Notably, HRD tumors harbored a high proportion of proinflammatory M1 macrophages (Figure 4C), which are involved in immune‐active related pathways, including chemokine signaling pathways, antigen processing and presentation, and cytokine‒cytokine receptor interactions (Figure 4D,E). Accordingly, the intercellular interactions between malignant epithelial cells and TAMs also validated more antigen presentation interactions in the HRD group (Figure 4F). Such results suggest that TAMs in the HRD group, especially the M1 subtype, act as antigen‐presenting cells that recognize tumor neoantigens, thereby recruiting more immune cells and creating an inflammatory environment. Conversely, HRP tumors were infiltrated with more inactive monocytes (Figure 4C) and M2 macrophages with severely impaired antigen‐presenting functions (Figure 4D), which indicated that insufficient tumor antigen signaling in the HRP group may fail to fully activate TAMs, allowing them to polarize into immunosuppressive M2 macrophages under the influence of an aberrant TIME.^[^ ^31^ ^]^ Therefore, we proposed anti–CSF1/CSF1R agonists as a potential therapeutic strategy for HRP tumors to inhibit macrophage infiltration and M2 differentiation.^[^ ^32^ ^]^

In summary, above results revealed distinct TMEs between the HRD and HRP groups, with antigen‐presenting M1 macrophages enrichment in HRD tumors and M2 macrophages enrichment in HRP tumors. This finding indicated divergent TAM differentiation trends and suggested that distinct immunotherapies customized to each subtype are needed. Importantly, anti‐CSF1/CSF1R agonists targeting immunosuppressive M2 TAMs may be an effective treatment modality for HRP tumors.

HGSTOC, High grade serous tubo‐ovarian carcinoma; HRP, Homologous recombination proficiency; HRD, Homologous recombination deficiency; TAM, Tumor‐associated macrophages; Qusage, Quantitative Set Analysis for Gene Expression; UMAP, Uniform manifold approximation and projection

### 2.5 Heterogeneous CD8+ and CD4+ T Cell Compartment in HRD and HRP Tumors

**Figure 5:**
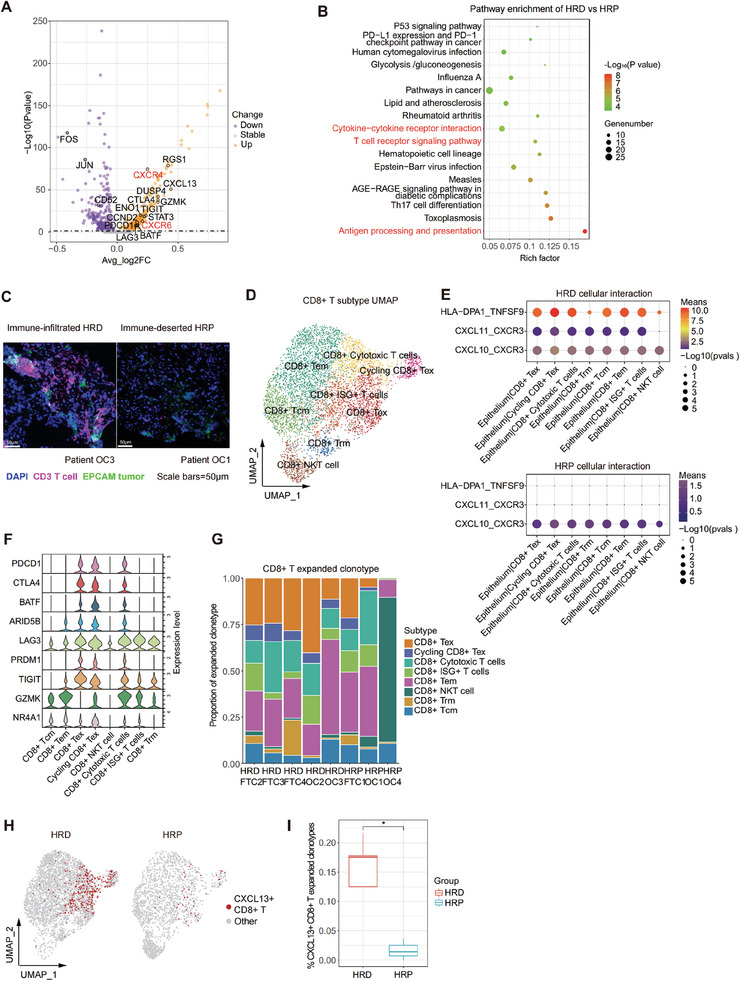
CD8+ T cells: clonal expanded tumor‐reactive T cells in HRD group versus bystander T cells in HRP group. A) A volcano plot demonstrating the differentially expressed genes of CD8+ T cell in HRD and HRP groups. The abscissa represented ‐log10(p value). P value was calculated by a Wilcoxon signed rank test. The ordinate represented average log2(fold change) of each cell. Purple, down regulated genes; orange, up regulated genes. B) A dot plot showing significant enriched pathways of CD8+ T cells in HRD and HRP tumors. P value was calculated by fisher exact test. C) Multiplexed IHC staining showing epithelial cells (EPCAM+, green) and T cells (CD3+, mauve) in representative samples of HRD and HRP group. Scale bar = 50 µM. D) A UMAP plot demonstrating CD8+ T cells reclustered into 8 subtypes. E) A cellphonedb dot plot of receptor‐ligand interactions between epithelial cell and CD8+ T cell subtypes in HRD (upper) and HRP (lower) groups. Dot size represented the p value of ligand‐receptor interaction. Dot color represented the means of ligand‐receptor interaction (Red, higher mean expression; purple, lower mean expression). F) A violin plot showing exhausted and cytotoxic signatures of CD8+ T cell subtypes. G) A column plot demonstrating the clonal expansion of CD8+ T cell subtypes in HRP and HRD samples. H) A UMAP plot showing tumor‐reactive CD8+ CXCL13+ T cells (clonal expansion ≥ 2) in HRD and HRP groups. Red, CD8+ CXCL13+ T cells; grey, other CD8+ T cells. I) A column plot showing tumor‐reactive CD8+ CXCL13+ T cells(clonal expansion ≥ 2) in HRD and HRP groups. P value was calculated by a Wilcoxon signed rank test. *P < 0.05.

Given that T cells are the major immune cell type that infiltrates tumors to mediate antitumor immunity in the TIME, we next analyzed the characteristics of CD8+ T cells and CD4+ T cells in the HRD and HRP groups. Of note, compared to those in the HRP group, CD8+ T cells in HRD tumors were characterized by high expression of chemokine receptor genes such as CXCR6 and CXCR4 (**Figure** **5**A) and were positively associated with cytokine‐cytokine receptor interactions, antigen processing and presentation, and the T‐cell receptor signaling pathway (Figure 5B). Similarly, CD4+ T cells in the HRD group received enhanced antigen presentation signals and were differentiated into effector T cells (Th1, Th2, Th17 cells) (Figure S5A, Supporting Information). Such results further supported our hypothesis that potent neoantigens from HRD tumors help recruit more T cells (CD8+ and CD4+ T cells) within the TIME than those from HRP tumors, and this finding was also validated by mIHC analysis (Figure 5C).

#### 2.5.1 Clonally Expanded Tumor‐Reactive T Cells in the HRD Group Versus Bystander T Cells in the HRP Group

To gain more insights into CD8+ T cell subsets in different functional states, we reclustered CD8+ T cells and grouped them into 8 subsets (Figure 5D; Figure S4A, Supporting Information): central memory T cells (Tcm cells) (IL7R, CCR7, TCF7), effector memory CD8+ T cells (Tem cells) (GZMK), tissue‐resident memory CD8+ T cells (Trm) (GNLY and XCL1), CD8+ Tex cells (PDCD1, CTLA4, HAVCR2, and TIGIT), cycling CD8+ Tex cells (MKI67, TOP2A, and PDCD1), CD8+ NKT cells (CX3CR1, FCGR3A, and FGFBP2), cytotoxic T cells (GZMK, PDCD1, and CTLA4), and CD8+ ISG+ T cells (ISG15, IFIT1, and IFIT3).^[^ ^19^, ^33^ ^]^

Next, we compared the infiltration of CD8+ T cells (clonal expansion ≥2) reflecting functionally active T cells between the HRD and HRP groups. Remarkably, recruited by potent antigen presentation signal stimulation (Figure 5E), the HRD group had a large proportion of CD8+ Tex cells and cycling CD8+ Tex cell clonotypes with high expression of cytotoxic signatures (GZMK) as well as exhausted markers (PDCD1, CTLA4, LAG3 and TIGIT),^[^ ^34^, ^35^ ^]^ which not only indicates their impaired antitumor effects but also, more importantly, highlights the tremendous potential of ICB therapy, specifically targeting CTLA4, PDCD1, LAG3, and TIGIT (Figure 5F,G), as promising therapeutic strategies for HRD tumors. For CD8+ T cells in HRP tumors, clonal expansion primarily involved functionally quiescent CD8+ Tcm cells and CD8+ Tem cells, suggesting that most CD8+ T cells in the HRP group were not fully stimulated due to insufficient tumor antigen presentation (Figure 5G). Therefore, enhancing the secretion and presentation of immunogenic neoantigens, such as through tumor vaccines or cytokine therapies, to activate immune cells, especially CD8+ T cells, may be a promising treatment strategy for HRP tumors.

While clonally expanded T cells are presumed to be more functionally active, the antigens targeted by such active T cells are not necessarily tumor antigens.^[^ ^34^ ^]^ Thus, we differentiated tumor‐reactive T cells from bystander T cells using the CXCL13 biomarker.^[^ ^21^ ^]^ Evidently, the potential tumor‐reactive T cells were predominantly cytotoxic T cells, Tex cells, and cycling Tex cells that received recruitment and antigen presentation signals (Figure 5H). Furthermore, we found that the HRD group had more abundant potential tumor reactive T cells than the HRP group (Figure 5I). Therefore, the combined analysis of T‐cell clonal expansion and function indicated that T cells in HRD tumors could recognize potent tumor antigen presentation signals and proliferate, whereas most clonally expanded T cells in HRP tumors were bystander T cells that recognize nontumor antigens. Such differentiation states of T cells were consistent with the immune‐active TIME of HRD tumors and the immune‐excluded TIME of HRP tumors.

HRP, Homologous recombination proficiency; HRD, Homologous recombination deficiency; UMAP, Uniform manifold approximation and projection; IHC, Immunohistochemistry

#### 2.5.2 Clonally Expanded Tregs in the HRD Group Versus Quiescent Tcm Cells in the HRP Group

**Figure 6:**
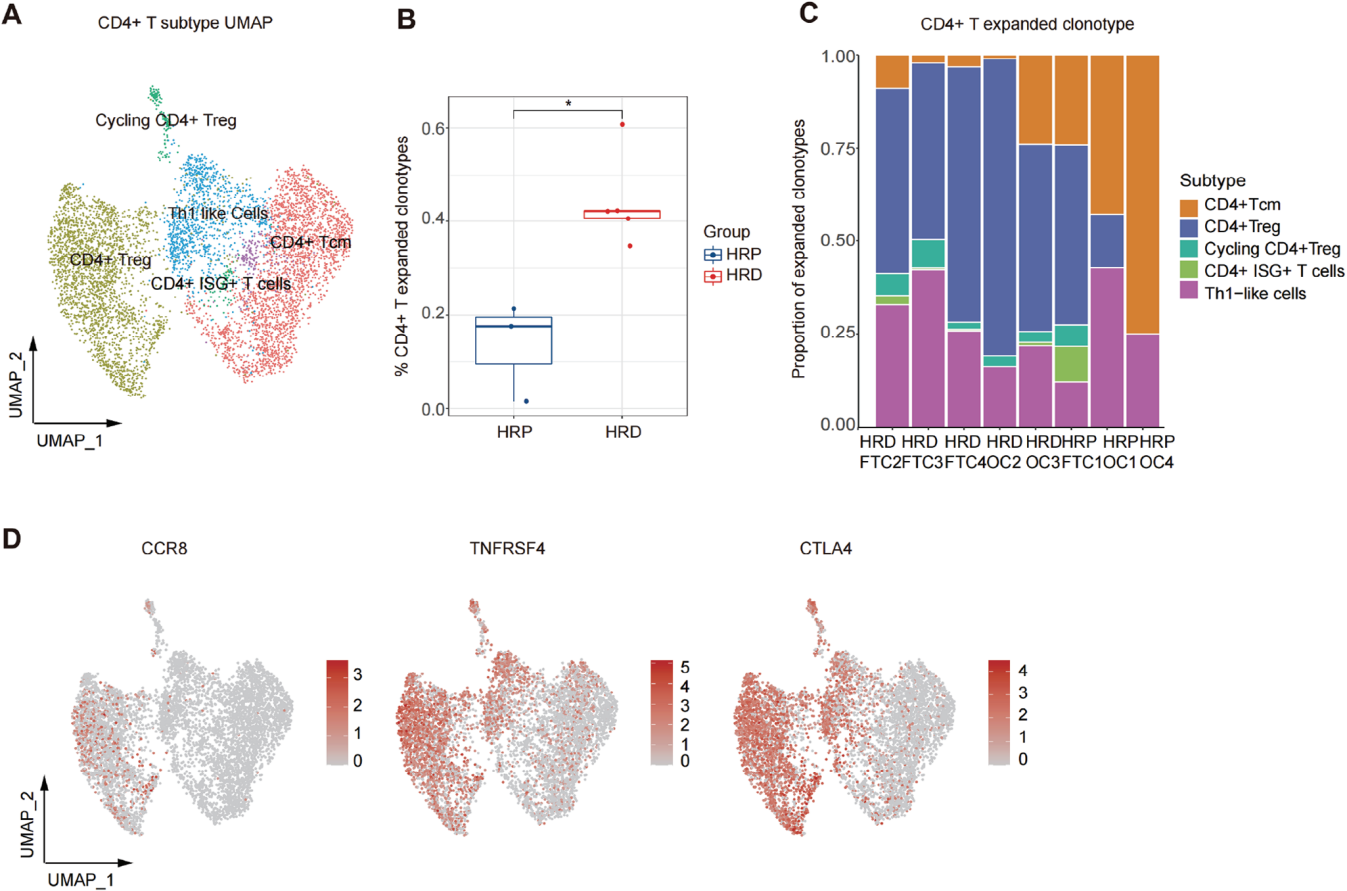
CD4+ T cells: massive clonal expanded Treg in HRD group versus quiescent Tcm cells in HRP group. A) A UMAP plot demonstrating CD4+ T cells reclustered into 5 subtypes. B) A boxplot showing clonal expansion of CD4+ T cell in HRD and HRP groups. P value was calculated by a Wilcoxon signed rank test. *P < 0.05. C) A column plot representing the proportion of CD4+ T cell subtypes (expanded clonotypes ≥ 2) in each sample. D) UMAP plots demonstrating the expression level of CCR8, TNFRSF4 and CTLA4 in CD4+ T cells. Bar color, represented the gene expression level (Red, higher expression; grey, lower expression).

We further reclustered CD4+ T cells into the following five subgroups on the basis of unsupervised clustering and marker gene expression.^[^ ^19^, ^33^ ^]^ (**Figure** **6**A; Figure S5C,D, Supporting Information): CD4+ Tcm cells (CCR7, TCF7), CD4+ Treg cells (FOXP3, CTLA4, TIGIT), cycling CD4+ Treg cells (MKI67, TOP2A), Th1‐like cells (CXCL13, IFNG), and CD4+ ISG+ T cells (ISG15, IFIT1, IFIT3).

Notably, CD4+ T cells in the HRD group had a higher degree of clonal expansion than those in the HRP group (Figure 6B). We also compared the infiltration of CD4+ T cells (clone expansion number ≥ 2) between the HRD group and HRP group and found that the HRD group harbored a large proportion of clone‐expanded CD4+ Tregs and cycling CD4+ Tregs (Figure 6C). Such results suggest that although many tumor‐reactive CD8+ T cells are recruited to combat HRD tumor cells, the infiltration of immunosuppressive Treg cells was also enhanced to hamper effective antitumor immune responses, which potentially explains the current poor response to immunotherapy. Therefore, targeting CD4+ Tregs could be an effective treatment approach to improve the immunotherapy response of patients with HRD tumors. Given that Treg cells exhibit robust expression of the chemokine receptor CCR8, which drives Treg recruitment, as well as TNFRSF4, which functions as a costimulatory molecule (Figure 6D), we proposed the following approaches: employing anti‐CCR8^[^ ^36^ ^]^ antibodies to mitigate Treg infiltration and stimulating TNFRSF4^[^ ^37^ ^]^ to counteract Treg‐mediated immunosuppression in HRD tumors. However, in the HRP group, mainly CD4+ Tcm cells underwent clonal expansion, which is consistent with the distribution of clonally expanded CD8+ T cells (Figure 6C). Therefore, we suggest that activating these quiescent bystander CD4+ T cells through tumor vaccines or cytokine therapies could be a therapeutic approach.

In summary, we found clonally expanded tumor‐reactive CD8+ T cells and clonally expanded Treg cells in the HRD group. In contrast, in the HRP group, we identified abundant quiescent bystander CD8+/CD4+ T cells. Moreover, we propose targeting exhausted T cells and immunosuppressive Tregs in the HRD groups and enhancing tumor antigen stimulation through approaches such as tumor vaccines or cytokine therapies in the HRP groups.

HRP, Homologous recombination proficiency; HRD, Homologous recombination deficiency; UMAP, Uniform manifold approximation and projection; Treg, Regulatory T cell; Tcm, Central Memory T cell

### 2.6 Clinical Insights: Potential Novel Therapeutic Strategies for HRD and HRP Groups

**Figure 7:**
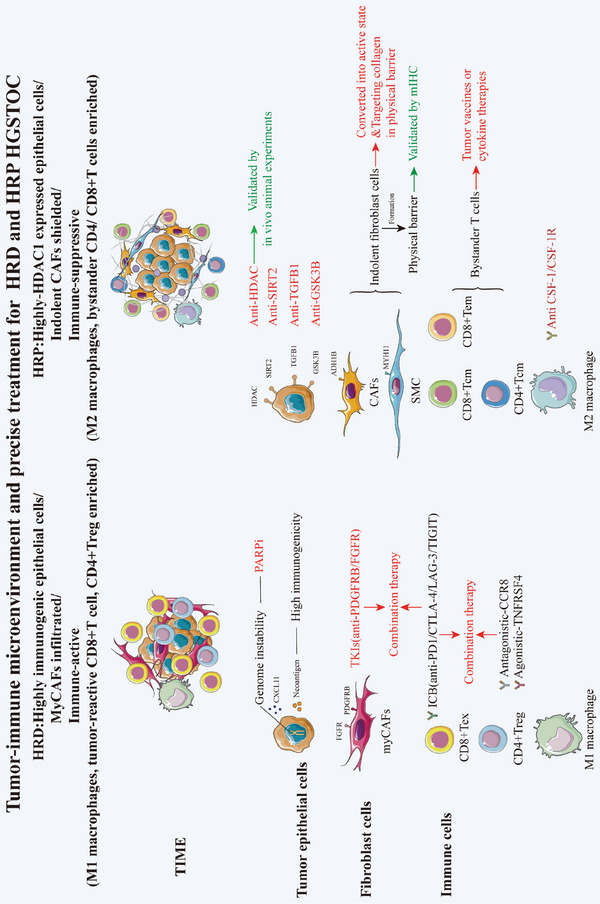
A summary diagram of tumor immune microenviroment and precise treatment for HRD and HRP HGSTOC patients.

Based on the above results regarding both structural cells (malignant epithelial cells, CAFs) and immune cells (macrophages, CD8+/CD4+ T cells), as well as the mIHC validaion conducted on 6 HRD samples and 6 HRP samples, which confirmed higher infiltration of CXCL13+ CD8+ T cells, CD4+ Treg cells and M1 macrophages in HRD, along with elevated HDAC1 expression in HRP tumors (Figure S6, Supporting Information), we aimed to propose precise treatment modalities customized to HRD and HRP patients (**Figure** **7**).

For HRD tumors, we proposed novel therapeutic modalities in addition to PARPis considering the presence of PARPi‐resistant patients within the HRD‐positive cohort. (1) CAF‐targeted therapies: TKIs constitute viable alternative treatment options for HRD tumors, given the abundance of FGFR1+PDGFRΒ+ myCAFs within the HRD TIME. (2) Treg cell‐targeted therapies: Anti‐CCR8 antibodies and anti‐CTLA4 antibodies to inhibit Treg infiltration and TNFRSF4 stimulation to inhibit Treg immunosuppression showed great promise given the substantial recruitment of immunosuppressive Tregs within the HRD TIME. (3) CXCL13+ CD8+ T‐cell‐targeted therapies: Considering the large number of CXCL13+ tumor‐reactive CD8+ T cells, restoring the function of these cells is of great importance. ICB drugs such as anti‐CTLA4/LAG3/TIGIT antibodies may be effective considering their high expression on tumor reactive T cells. Finally, combination therapies targeting both CXCL13+ CD8+ T cells and CAFs/Treg cells could also be considered to overcome the clinical challenges of acquired resistance in the HRD population and the limited efficacy of PARPi and anti‐PDCD1 combination therapy.

For HRP tumors, (1) epithelial cell‐targeted therapy: Given the high HDAC1 expression in the epithelial cells of HRP tumors and the remarkable efficacy of HDACis in tumor‐bearing mouse models, we proposed HDACis as a potential treatment. Furthermore, we also suggested targeting certain upregulated molecules in HRP tumors, including GSK3B, TGFB1 and SIRT2, which are associated with poor prognosis. (2) CAF‐targeted therapies: Transcriptomic analysis combined with mIHC validation suggested that the large population of “indolent” CAFs (ADH1B+ CAFs and MYH11+ SMCs) formed robust physical barriers within the TIME, impeding immune cell infiltration and potentially blunting the effects of adoptive immunotherapies. Therefore, diminishing “indolent” CAFs or reprogramming them into immune‐active CAFs might enhance the efficacy of immunotherapies for HRP patients, which merits further preclinical investigation. (3) Macrophage‐targeted therapies: We propose blocking CSF1/CSF1R to inhibit macrophage infiltration and M2 polarization in the HRP group. (4) T cell‐targeted therapies: Enhancing tumor antigen secretion and presentation, such as through tumor vaccines or cytokine therapies, could effectively activate the large population of dormant immune cells (CD8+/CD4+ bystander T cells) within the HRP TIME.

Schematic representation of distinct tumor immune microenvironment and novel potential targets on epithelial cells, fibroblast cells and immune cells uniquely to HRD and HRP HGSTOC patients.

HRD, homologous recombination deficient; HRP, homologous recombination proficient; HGSTOC, high‐grade serous tubo‐ovarian cancer.

## 3 Discussion

HGSTOC is a highly aggressive gynecological malignancy with a poor prognosis and a high risk of recurrence. HGSTOC can be stratified into HRD and HRP subtypes based on HR status.^[^ ^5^ ^]^ Although the therapeutic potential of PARPis and anti‐PDCD1 antibodies in managing HGSTOC has been observed, their clinical value within HRD and HRP cohorts is restricted by 1) PARPi resistance and ineffective anti‐PDCD1 therapy in HRD patients and 2) the ineffectiveness of PARPis within the majority of HRP patients, coupled with an absence of efficacious targeted or immunological interventions.^[^ ^7^, ^8^, ^9^ ^]^ To address this clinical dilemma, elucidating the intricacies of the TME and developing novel and efficacious target‐based therapeutic modalities customized to HRD and HRP patients are urgently needed. In this study, we generated a single‐cell transcriptional atlas of five HRD and three HRP HGSTOC samples by scRNA‐seq and TCR‐seq. Notably, we discovered that HRD tumors were highly enriched in immunogenic epithelial cells, FGFR1+PDGFRβ+ myCAFs, M1 macrophages, tumor reactive CD8+ T cells, and CD4+ Tregs, while HRP tumors were highly enriched in HDAC1‐expressing epithelial cells, indolent CAFs, M2 macrophages, and bystander CD4+/CD8+ T cells. For HRD patients, we proposed potential therapies to overcome the treatment challenges posed by PARPi resistance and the limited clinical benefit achieved by PARPi and anti‐PDCD1 combination therapy. (1) We discovered that targeting FGFR1+PDGFRβ+ CAFs with TKIs might be efficient. (2) We discerned that the combination of reactivating CD8+ Tex cells with anti‐PDCD1/CTLA4/LAG‐3/TIGIT antibodies and targeting Tregs with antagonistic CCR8/CTLA‐4 and agonistic TNFRSF4 or myCAFs with TKIs as effective therapies. For HRP patients, we have identified several potential effective strategies: (1) We proposed that HDACis could serve as effective therapies, which has been validated by animal experiments. (2) We identified the presence of a physical immune barrier formed by “indolent” CAFs surrounding tumor cells that promotes an immunosuppressive TME. The presence of these cells was validated by mIHC staining, and they could serve as novel targets. (3) We suggest that targeting M2 macrophages with anti–CSF1/CSF1R agonists and targeting bystander T cells using tumor vaccines or cytokine treatments might play a crucial role in improving the efficacy of HRP treatment.

To date, PARPis targeting epithelial cells remain the primary maintenance therapy for HGSTOC patients after standard first‐line treatment, but resistance and non‐responsiveness still occur in HRD and HRP patients,^[^ ^6^ ^]^ which highlights the urgency to not only develop methods that amplify PARPi responsiveness for HRD patients but also to identify novel therapeutic targets for HRP patients. In this study, we initially explored the unique biological characteristics of epithelial cells based on HR status. In HRD patients, epithelial cells remained as genome‐unstable cells, suggesting a potential high neoantigen load, which is correlated with increased immunogenicity.^[^ ^38^ ^]^ In HRP patients, epithelial cells were distinctly characterized by high expression of HDAC1, SIRT2, TGFB1, and GSK3B, which are correlated with poor prognosis in HGSTOC, suggesting that these factors could be novel potential therapeutic targets. Notably, as verified by in vivo experiments, HDACis exerted more effective antitumor effects both when used as monotherapy and when combined with PARPis for HRP patients. In summary, we not only revealed the distinct features of epithelial cells in HRD (high immunogenicity) and HRP (high HDAC1 expression) tumors but also proposed HDACis as novel therapeutic strategy for HRP tumors.

Increasing evidence has underscored the importance of CAFs in tumor progression and prognosis.^[^ ^39^ ^]^ However, there has been limited exploration into the role of CAFs in ovarian cancer and their potential implications for therapeutic strategies. In the present study, we observed that FGFR1+PDGFRβ+ myCAFs were enriched in HRD tumors, contributing to ECM remodeling and tumor cell proliferation, which highlights the potential of TKIs as an effective therapeutic approach by disrupting the signaling pathways involved in tumor growth in myCAFs, thereby counteracting their pro‐proliferative effects.^[^ ^40^, ^41^ ^]^ Remarkably, we have, for the first time, identified and defined a subset of “indolent” CAFs (ADH1B+ CAFs and MYH11+ SMCs) in HRP tumors, which is distinguished by quiescent functionality, limited intercellular interactions, and the propensity to establish a physical barrier encircling neoplastic cells, whose presence was validated by mIHC staining. This physical barrier effectively impedes the infiltration of immune cells into tumor cells, thereby fostering an immunosuppressive milieu. Importantly, given that active ADH1B+ CAFs, which can express genes responsible for T‐cell chemotaxis and retention,^[^ ^24^ ^]^ tend to remain inactive and form a type IV collagen barrier that impedes T‐cell invasion, we suggested that converting inactive ADH1B+ CAFs into an active state may facilitate T‐cell invasion and that targeting the physical collagen barrier to reverse the immunosuppressive TME might be effective in HRP patients which still required further exploration. Consequently, our findings highlighted the enrichment of FGFR1+PDGFRβ+ myCAFs in HRD tumors and identified the unique physical barrier formed by indolent CAFs obstructing immune cell infiltration in HRP tumors. These observations provide insights into potential therapies targeting myCAFs by TKIs for HRD and targeting “indolent” CAFs for HRP patients.

The existing immunotherapy regimens have shown lackluster clinical outcomes in HGSTOC patients, and effective immune targets still need to be identified.^[^ ^10^ ^]^ In the present study, we illustrated the intricate immune landscape in detail and revealed potential immune targets specifically for HRD and HRP tumors. For HRD tumors, our results indicated an immune‐active tumor microenvironment with enrichment of M1 cells, CD8+ cytotoxic T cells and CD8+ Tex cells. Notably, we discovered that an abundance of tumor‐reactive CXCL13+CD8+ Tex cells highly expressed cytotoxic‐related genes (GZMK) and exhausted‐related genes (PDCD1/CTLA4/LAG3/TIGIT), indicating the clinical potential of ICB, especially anti‐PDCD1/CTLA4/LAG3/TIGIT antibodies, in reactivating CXCL13+CD8+ Tex cells to combat tumor cells. Interestingly, although we observed immune‐active tumor‐reactive CD8+ T cells within the HRD TIME, immune‐suppressive CD4+ Tregs were also enriched. This paradoxical observation may explain the poor clinical response and unfavorable prognosis seen in some HRD patients who received anti‐PDCD1 monotherapy or PARPi plus anti‐PDCD1 combination therapy. The substantial suppressive impact exerted by Tregs on the immune response is likely a key contributor.^[^ ^42^ ^]^ Therefore, targeting Tregs by antagonistic CCR8/CTLA‐4 and agonistic TNFRSF4 to alleviate Treg‐mediated immune suppression within the TIME might further enhance the efficiency of ICB (anti‐PDCD1/CTLA4/LAG3/TIGIT) therapies and PARPi‐anti‐PDCD1 combination therapy. For HRP tumors, by contrast, an immune‐suppressive microenvironment with enrichment of inactive bystander T cells and M2 macrophages was observed, underscoring a milieu that hinders immune effector activity. Therefore, to improve the immunotherapy efficiency in HRP tumors, inactive bystander T cells could be converted into an active state to exert an antitumor effect by tumor vaccines or cytokine therapies such as IL‐15 therapy.^[^ ^43^ ^]^ In parallel, converting M2 macrophages into immunostimulatory M1 macrophages and eliminating TAMs by targeting CSF1/CSF1R may also be effective.^[^ ^44^ ^]^ In summary, we clarified the immune‐active (enrichment of M1 macrophages, tumor reactive CD8+ T cells, and CD4+ Tregs) microenvironment in HRD tumors and the immune‐suppressive (enrichment of M2 macrophages and bystander CD4+/CD8+ T cells) microenvironment in HRP tumors. For HRD tumors, we suggest the combined administration of anti‐CCR8/CTLA‐4 or TNFRSF4 stimulator targeting Tregs and ICB (anti‐PDCD1/CTLA4/LAG‐3/TIGIT) to restore the activity of CXCL13+CD8+ Tex cells. For HRP tumors, we proposed activating bystander T cells by tumor vaccines or cytokine therapies and targeting M2 macrophages by anti‐CSF1/CSF1R antibodies.

In conclusion, we demonstrated a distinct single‐cell transcriptomic atlas of HRD and HRP tumors using scRNA‐seq and scTCR‐seq, covering epithelial cells, fibroblasts, and immune cells, within different HRD statuses determined by HRD score calculation. Importantly, we found that HRD tumors were highly enriched in immunogenic epithelial cells, infiltrated myCAFs, M1 macrophages, tumor reactive CD8+ T cells, and CD4+ Tregs, while HRP tumors were highly enriched in epithelial cells expressing HDAC1, indolent CAFs, M2 macrophages, and bystander CD4+/CD8+ T cells. Of note, for HRD patients, in addition to PARPis, we suggested that targeting FGFR1+PDGFRΒ+ myCAFs by TKIs, targeting CD8+Tex by ICB therapy (anti‐PDCD1/CTLA4/LAG‐3/TIGIT) and targeting Tregs by CCR8 or CTLA‐4 antagonists/ TNFRSF4 agonists may be effective. We proposed that the co‐administration of targeting Tex cells and CAFs/Tregs might improve the responsiveness to PARPis or PARPi‐anti‐PDCD1 combination therapies. Remarkably, for HRP patients, we proposed that targeting epithelial cells with HDACis holds promise as a potentially efficacious treatment with validation in animal experiments. Additionally, we revealed a physical barrier crafted by indolent CAFs (ADH1B+ and MYH11+ CAFs) encircling malignant cells and fostering an immunosuppressive microenvironment, which was validated by mIHC staining. Moreover, we have proposed the notion that anti‐CSF‐1/CSF‐1R targeting M2 macrophages, tumor vaccines or cytokine therapies targeting bystander T cells might be effective for HRP tumors. In summary, these findings not only reveal unique TMEs but also provide novel insights for developing effective clinical therapies customized for HRD and HRP patients.

## 4 Experimental Section

### Human Specimens

A total of 8 high‐grade serous tubo‐ovarian cancer specimens were collected at the Obstetrics and Gynecology Hospital of Fudan University, Shanghai, China between 2021 and 2022. All patients met the following criteria: (1) pathological diagnosis of high‐grade serous fallopian tube carcinoma or high‐grade serous ovarian cancer; (2) underwent primary cytoreductive surgery; (3) no history of other cancers; (4) no preoperative radiotherapy or chemotherapy. This study was approved by the Ethics Committee of the Obstetrics and Gynecology Hospital of Fudan University (2022‐106), and written informed consent was obtained from all participants.

### Single‐Cell Dissociation

Single‐cell RNA sequencing was performed by experienced personnel in the laboratory of NovelBio Co., Ltd. The tissue samples were surgically removed and kept in MACS Tissue Storage Solution (Miltenyi Biotec) until processing. The tissues were processed as follows: Briefly, samples were first washed with phosphate‐buffered saline (PBS), minced into small pieces (approximately 1 mm^3^) on ice, and enzymatically digested with a tumor dissociation kit (Miltenyi Biotec) for 30 min at 37 °C with agitation. After digestion, the samples were sieved through a 70 µm cell strainer and centrifuged at 300 g for 5 min. After removal of the supernatant, the pelleted cells were suspended in red blood cell lysis buffer (Miltenyi Biotec) to lyse red blood cells. The cells were then washed with PBS containing 0.04% BSA, and the cell pellets were re‐suspended in PBS with 0.04% BSA before being re‐filtered through a 35 µm cell strainer. The dissociated single cells were stained with AO/PI for viability assessment using a Countstar Fluorescence Cell Analyzer.

### Single‐Cell Sequencing

Single‐cell RNA St The scRNA‐Seq libraries and V(D)J libraries were generated using the 10X Genomics Chromium Controller and Chromium Single Cell 5′ Library & Gel Bead Kit, along with the V(D)J Enrichment Kit (10X Genomics, Pleasanton, CA). Briefly, cells were concentrated to 1000 cells µL^−1^ and loaded into each channel to generate single‐cell Gel Bead‐In‐Emulsions (GEMs). This resulted in the expected mRNA barcoding of approximately 5000 single cells for each sample. After the reverse transcription step, the GEMs were broken and barcoded cDNA was purified and amplified. The amplified barcoded cDNA was used to construct 5′ gene expression libraries and T cell receptor (TCR) enriched libraries. For the 5′ library construction, the amplified barcoded cDNA was fragmented, A‐tailed, ligated with adaptors, and index PCR amplified. For the V(D)J library, human TCR V(D)J sequences were enriched from the amplified cDNA followed by fragmentation, A‐tailing, adaptor ligation, and index PCR amplification. The final libraries were quantified using the Qubit High Sensitivity DNA Assay (Thermo Fisher Scientific) and the size distribution was determined using a High Sensitivity DNA chip on a Bioanalyzer 2200 (Agilent). All libraries were sequenced on an Illumina sequencer (Illumina, San Diego, CA) using 150 bp paired‐end sequencing.

### Survival Analysis

To evaluate the prognostic effects of HDAC1, SIRT2, GSK3B and TGFB1 expression in epithelial cells treated with histone deacetylase (HDAC) inhibitors versus histone retaining proteins (HRPs), gene expression data along with corresponding clinical survival information and HDAC inhibitor response status were obtained from The Cancer Genome Atlas (TCGA) ovarian cancer (OV) cohort. These data were publicly available from the TCGA database (https://portal.gdc.cancer.gov). When examining the effects of these drug target genes on patient survival, their relative expression levels were represented as transcripts per million (TPM) values. For all survival analyses, patients were stratified into high and low expression groups using optimal cutoff points. Kaplan‐Meier survival curves were generated using the R statistical software version 4.0.3, with the survminer and survival packages. P‐values were calculated using the log‐rank test to compare survival distributions between high and low expression groups.

### ScRNA‐Seq Data Processing

ScRNA‐seq data analysis was performed by NovelBio Co., Ltd. Using the NovelBrain Cloud Analysis Platform (www.novelbrain.com). Fastp was applied^[^ ^45^ ^]^ with default parameters to filter out adaptor sequences and remove low‐quality reads, generating clean data. The feature‐barcode matrices were then obtained by aligning reads to the human genome (GRCh38 Ensemble, version 104) using CellRanger v5.0.1. Down sampling analysis was applied across sequenced samples based on the number of mapped barcoded reads per cell, yielding the aggregated matrix. Cells containing over 200 and below 10000 expressed genes and less than 20% mitochondrial UMI rate passed quality control filtering, and mitochondrial genes were removed from the expression table. The Seurat package (version 3.1.4, https://satijalab.org/seurat/) was used for cell normalization and regression based on the UMI counts and percent mitochondria rate of each sample to obtain scaled data. PCA was performed on the scaled data using the top 2000 highly variable genes. The top 10 principal components were then used to construct UMAP projections. Graph‐based clustering was utilized on the top 10 principal components to obtain unsupervised cell clusters. Marker genes for each cluster were identified using the FindAllMarkers function with a Wilcoxon rank sum test under the following criteria: 1) lnFC > 0.25; 2) p‐value < 0.05; 3) min.pct > 0.1. To identify cell subtypes in more detail, clusters of the same cell type were selected for re‐analysis by tSNE, graph‐based clustering, and marker identification.

### Pseudo‐Time Analysis

Single‐cell trajectory analysis was performed using Monocle2 (http://cole‐trapnell‐lab.github.io/monocle‐release/) with the DDRTree method and default parameters. Prior to Monocle analysis, marker genes from the Seurat clustering results and raw expression counts for filtered cells were selected. Pseudo‐time analysis was applied, followed by branch expression analysis modeling (BEAM) to identify genes associated with branch fate determination.

### CytoTRACE

CytoTRACE was used^[^ ^45^ ^]^ to predict the stemness and developmental potential based on the number of expressed genes per cell. Generally, this algorithm predicts cell differentiation states from scRNA‐seq data based on the negative correlation between the number of expressed genes per cell and transcriptional diversity. CytoTRACE was used as a complement to the pseudotime trajectory analysis from Monocle2 to predict the stemness per cell.

### Cell Communication Analysis

To systematically analyze cell‐cell communication molecules, cell communication analysis was applied using CellPhoneDB,^[^ ^46^ ^]^ a public repository of ligands, receptors and their interactions. Membrane, secreted and peripheral proteins were annotated for clusters at different time points. Significant mean and cell communication significance (p‐value < 0.05) were calculated based on the cell subtype annotation for each cell and the cell count matrix obtained through Seurat.

### Differential Gene Expression Analysis

To identify differentially expressed genes among samples, the function FindMarkers with wilcox rank sum test algorithm was used under following criteria: 1. lnFC > 0.25; 2. P value < 0.05.

### Go Analysis

Gene ontology (GO) analysis was performed to facilitate elucidating the biological implications of marker genes and differentially expressed genes.^[^ ^47^ ^]^ The GO annotations were downloaded from NCBI (http://www.ncbi.nlm.nih.gov/), UniProt (http://www.uniprot.org/) and the Gene Ontology (http://www.geneontology.org/). Fisher's exact test was applied to identify the significant GO categories and FDR was used to correct the p‐values.

### Pathway Analysis

Pathway analysis was used to find out the significant pathway of the marker genes and differentially expressed genes according to KEGG database.^[^ ^48^ ^]^ The Fisher's exact test was turn to select the significant pathway, and the threshold of significance was defined by P‐value and FDR.

### QuSAGE (Quantitative Set Analysis for Gene Expression)

In order to quantify gene sets activity, the R package QuSAGE^[^ ^49^ ^]^ was used as described for gene set enrichment analysis to achieve the enrichment status and enrich significance of gene sets. QuSAGE accounted for inter‐gene correlations and corrected these correlations by improving the estimation of the variance inflation factor from expression data. This method is available as an R package to visualize the results as heatmap plots.

### Genomic Scar Analyses (GSA)

Genomic Scar Analyses (BGI genomics, China) evaluates homologous recombination deficiency by detecting the HRD score at the genome level, germline and somatic variation of 68 HRR pathway genes and fragment rearrangement of BRCA1/2. HRD score is obtained by adding the loss of heterozygosity (LOH) score, the telomeric allelic imbalance (TAI) score and the large‐scale state transitions (LST) score then corrected by tumor purity and ploidy.^[^ ^50^ ^]^ LOH was calculated using the number of LOH regions that were larger than 15 MB and less than the entire chromosome length.^[^ ^51^ ^]^ Largescale state transitions (LST) were large insertions and deletions of chromosomes breaks of at least 10 mb that cause copy number changes.^[^ ^52^ ^]^ Telomeric allelic imbalance (TAI) was defined as the number of chromosomal segments with allelic copy number imbalance that extend to the subtelomere but do not exceeding the centromere.^[^ ^50^ ^]^ The HRD status and HRR gene variation of each patient was shown in Table S1 (Supporting Information).

### In Vivo Murine Tumor Models

All animal experiments were performed under approval of the Fudan University Animal Care and Use Committee. Female BALB/c‐nu mice (5‐6 weeks old) were purchased from the Laboratory Animal Center of the Shanghai Institutes for Biological Sciences and housed in a specific pathogen‐free (SPF) environment. SKOV3 cell lines were obtained from the Chinese Academy of Sciences Shanghai Cellular Library and cultured in RPMI‐1640 medium (Thermo Fisher Scientific supplemented with 50 IU mL^−1^ penicillin G, 50 mg mL^−1^ streptomycin, 4 mM GlutaMAX, and 10% FBS (Thermo Fisher Scientific) at 37 °C with 5% CO2. To establish ectopic tumors, 5 × 10^6^ SKOV3 cells (in 100 µL phosphate buffered saline) were injected subcutaneously into the right shoulder of BALB/c‐nu mice (denoted as Day 0). When tumors reached approximately 50 mm^3^ in size, the tumor‐bearing mice were randomly divided into four groups (Day 5). For PARPi or HDACi treatment, mice received either 1 mg kg^−1^ TSA (Abmole) daily by intraperitoneal injection, 50 mg kg^−1^ Olaparib (Abmole) daily by oral gavage, or a combination of both for 2 weeks. Control mice received normal saline on a similar schedule. Tumors were measured every 2–4 days using calipers and tumor volumes calculated as V = (L × W^2^)/2, where V = volume (mm^3^), L = length (mm), and W = width (mm). To establish intraperitoneal xenograft model, 7 × 10^6^ SKOV3 cells (in 100 µL phosphate buffered saline) were injected internationally into the BALB/c‐nu mice and received the same treatment as subcutaneous xenograft model.

### Multiplexed Immunohistochemistry (mIHC) Staining

To elucidate the spatial localization of EPCAM+epithelial, ADH1B+CAFs, MYH11+CAFs, CD3+T cells, CD4+Treg, CXCL13+CD8+T, CD68+CD86+M1 cells, multiplexed immunohistochemistry staining TSA 7‐color kit (abs50015‐100T, Absinbio, Shanghai) and heterogeneous immunofluorescence double staining was performed. The slides were incubated with blocking diluent antibody at room temperature for 10 minutes followed by incubation of primary antibodies overnight at 4 °C and then conjugated to anti‐rabbit or anti‐mouse horseradish peroxidase‐conjugated (HRP) secondary antibody (abs50015‐02, Absinbio, Shanghai) for 10 minutes. Then, the sections were treated with a fluorophore (tyramide signal amplification plus working solution), followed by heat‐treatment using a microwave. Each slide was then treated with 2 drops of DAPI, washed in distilled water, and manually coverslipped. Slides were air dried, and take pictures with Pannoramic MIDI II (3DHISTECH). Images was analyzed using Indica Halo software. The primary antibodies used were anti‐CD3 (Cell Signaling Technology Cat.No. 85 061, 1:200), anti‐ADH1B(Affinity Biosciences Cat.No. DF12809 1:200), anti‐MYH11 (Abcam, Cat.No. ab133567, 1:500) and anti‐EPCAM (Cell Signaling Technology, Cat.No. 14452S, 1:500) antibody (EPCAM was a transmembrane glycoprotein identified as a tumor specific antigen,^[^ ^53^ ^]^) anti‐CD4 (Proteintech, Cat.No.67786, 1:500), anti‐Foxp3 (Servicebio, Cat.No. GB112325‐100, 1:900), anti‐CD8 (Abcam, Cat.No.AB17147, 1:100), anti‐CXCL13(Abcam, Cat.No.AB246518, 1:1000), anti‐CD68 (Servicebio, Cat.No.GB113150, 1:2000) anti‐CD86 (Proteintech, Cat.No. 26 903, 1:100).

### Immunohistochemistry (IHC)

For IHC analysis, an anti‐HDAC1 primary antibody (Affinity, AF6433, 1:100 dilution) was used. Tissue specimens were deparaffinized, rehydrated, and subjected to antigen retrieval in boiling citrate buffer for 3 minutes. Slides were then blocked in 3% BSA for 30 minutes, incubated with primary antibodies at 4 °C overnight, washed with Tris‐buffered saline containing 0.1% Tween 20 (TBS‐T), and subsequently incubated with an HRP‐labeled rabbit secondary antibody for 50 minutes. A DAB kit was used to visualize the staining. Images of the IHC were acquired using a microscope with 10x and 20x objectives. IHC staining was scored based on the percentage of positive area and intensity as follows: 0, no staining; 1, <10% positive with moderate or strong intensity; 2, 10–50% positive with moderate or strong intensity; 3, >50% positive with moderate intensity; and 4, >50% positive with strong intensity.

### Flow Cytometry

To detect the percentage of M1 cells in peritoneal lavage fluid, flow cytometry was conducted. The cells were extracted from the peritoneal lavage fluid, then incubated in PBS with the fixable viability dye (BD, 564 406) before antibody staining. Prior to surface staining with antibodies, Fc gamma receptors were blocked by incubating cells with anti‐CD16/CD32 antibodies (BD, 553 141). Thereafter, cells were incubated with following primary antibodies: CD45(eBioscience, 47‐0451‐82), CD11b(BD,101 255), F4/80(BD, 123 113), CD86(159 203) diluted in FACS buffer (DPBS + 2% FCS) for 15 min. All samples were run on a CytoFLEX platform (Beckman Coulter) and analyzed using FlowJo version 10.8 software (BD Biosciences). Flow cytometry gating strategy for identifying M1 cells were shown in Figure S2E (Supporting Information).

### Statistical Analysis

All statistical analysis was conducted using Prism 8.0 (GraphPad Software). All graphs depict mean ± SEM unless otherwise indicated. Statistical significances were denoted as not significant (ns; P > 0.05), *P < 0.05, **P < 0.01, ***P < 0.001, ****P < 0.0001. The numbers of experiments were noted in figure legends. To assess the statistical significance between two groups, Wilcoxon signed rank test, Mann‐Whitney test, Welch's *t*‐test, fisher exact test, unpaired *t*‐test. For Kaplan–Meier overall survival analysis, p value was calculated by log‐rank test was used. For comparison between multiple groups, differences were tested by one‐way ANOVA or two‐way ANOVA followed by Tukey's multiple comparison tests.

## Conflict of Interest

The authors declare no conflict of interest.

## Author Contributions

J.Q., T.R., and Q.L. contributed equally to this work. J.Q,. X.Q., and K.H. initiated the project and designed and supervised the research plan. T.R., Q.L., Q.J., T.W., L.C., X.H., and W.Y. performed stimulation experiments under the supervision of K.H. T.R., Q.L., and X.Q. wrote the manuscript. Q.J., T.W., L.C., W.Y. and X.H. designed and performed the supporting experiments. Q.J., T.W., L.C., and W.Y. made significant revisions in language to the manuscript. The order of first authorship was determined by contribution to project design. All authors edited and approved the manuscript. K.H was responsible for the overall content as guarantor.

## Supporting information

Supporting information

## Data Availability

The data that support the findings of this study are available from the corresponding author upon reasonable request.
