# Supplementary material for: Dissecting the Distinct Tumor Microenvironments of HRD and HRP Ovarian Cancer: Implications for Targeted Therapies to Overcome PARPi Resistance in HRD Tumors and Refractoriness in HRP Tumors
Source: Adv Sci (Weinh). 2024 Aug 13;11(38):2309755. doi: 10.1002/advs.202309755 (PMC11481286; doi:10.1002/advs.202309755)
Supplement: Supplementary file 1 — Supporting information [file ADVS-11-2309755-s001.pdf]

ADVANCED SCIENCE
Open Access

Supporting Information

for *Adv. Sci.*, DOI 10.1002/advs.202309755

Dissecting the Distinct Tumor Microenvironments of HRD and HRP Ovarian Cancer: Implications for Targeted Therapies to Overcome PARPi Resistance in HRD Tumors and Refractoriness in HRP Tumors

Junjun Qiu, Tingting Ren, Qinqin Liu, Qian Jiang, Tong Wu, Leong Chi Cheng, Wenqing Yan, Xinyu Qu, Xiao Han* and Keqin Hua**

Supporting Information

Dissecting the Distinct Tumor Microenvironments of HRD and HRP Ovarian Cancer: Implications for Targeted Therapies to Overcome PARPi Resistance in HRD Tumors and Refractoriness in HRP Tumors

Junjun Qiu[†], Tingting Ren[†], Qinqin Liu[†], Qian Jiang, Tong Wu, Leong Chi Cheng, Wenqing Yan, Xinyu Qu[*], Xiao Han[*], Keqin Hua[1,2,]*

Table S1

Clinical information, HRD score and HRR (Homologous Recombination Repair) gene variation of each sample

Patient ID	Age (year)	Histologic type	HRD status	HRD score	BRCA1/2 variation	68 HRR gene germline mutation	68 HRR gene somatic mutation
OC-1	60	left ovary high grade serous carcinoma	Negative	17.5	wild type	FANCD2(III)	TP53(II), NF1(II)
OC-2	41	bilateral ovary high grade serous carcinoma	Positive	59.76	wild type	TP53(III), CDK12(III), FANCA(III)	TP53(II)
OC-3	57	ovary high grade serous carcinoma	Positive	24.21	BRCA2 c.517-1G>A(I)	ERCC5(III)	BRCA2(I),TP53(II)
OC-4	57	ovary high grade serous carcinoma	Negative	30.39	wild type	PALB2(III), RAD51(III)	TP53(III)
FTC-1	55	fallopian tube fimbria high grade serous carcinoma	Negative	<1	wild type	CFTR(III), FANCI(III), FANCM(III)	TP53(II)
FTC-2	63	bilateral fallopian tube high grade serous carcinoma	Positive	61.47	BRCA2 p.Tyr1655*(c.4965delC)(I)	BRCA2(I), ERCC4(III), MUS81(III), XRCC2(III)	TP53(II), CHEK2(II), PALB2(II), RAD54L(III), BRCA2(III), MLH1(III)
FTC-3	51	fallopian tube fimbria high grade serous carcinoma	Positive	50.54	wild type	BRCA1(III), EME1(III), FANCL(III)	TP53(II)
FTC-4	69	fallopian tube high grade	Positive	57.92	wild type	SMARCA4(III)	TP53(II)

	serous carcinoma

Note: Germline and somatic variants are classified according to ACMG Guidelines (Richards (2015): tier I-variants with strong clinical significance; tier II- variants with potential clinical significance; tier III-variants of uncertain clinical significance(VUS)

Table S2

Differentially expressed CXCL8, CXCL10, CXCL11 of epithelial cells between HRD and HRP patients

Gene	P value	log2FC	HRD	HRP	P value adj	upregulated/downregulated
CXCL8	3.60E-93	0.4825494	0.214	0.087	1.04E-88	upregulated
CXCL11	3.71E-72	0.42890372	0.102	0.025	1.07E-67	upregulated
CXCL10	4.87E-37	0.64432696	0.225	0.144	1.40E-32	upregulated

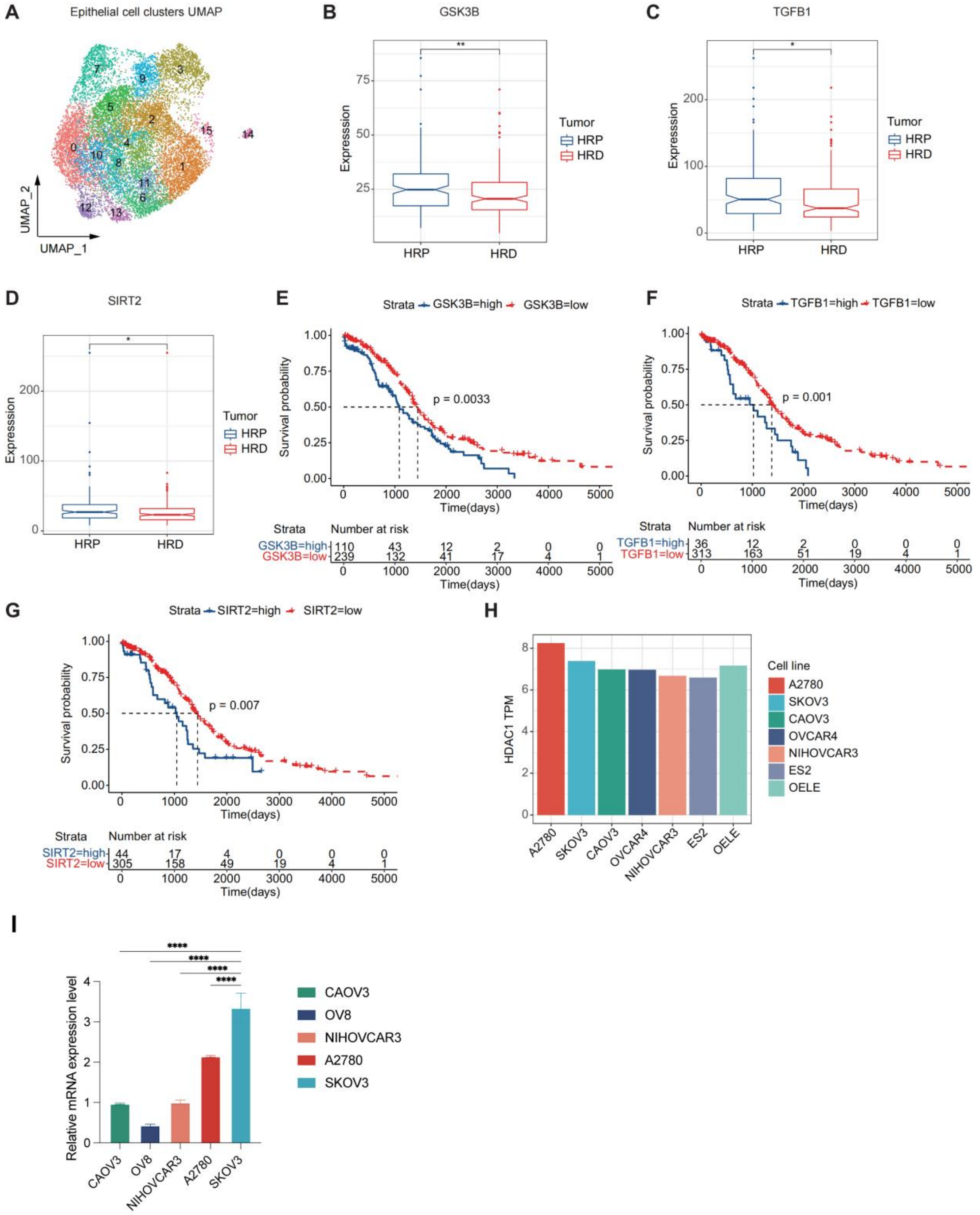

Figure S1. HDAC inhibitors demonstrate promising therapeutic effects for HRP

tumors.

(A) A UMAP plot demonstrating epithelial cells reclustered into 16 subclusters.

(B) A box plot demonstrating the expression of GSK3B in HRP and HRD tumors.

(C) A box plot demonstrating the expression of TGFB1 in HRP and HRD tumors.

(D) A box plot demonstrating the expression of SIRT2 in HRP and HRD tumors.

(E)Kaplan–Meier overall survival curves illustrating the prognostic value of GSK3B gene expression, validated in TCGA HGSTOC cohorts.

(F)Kaplan–Meier overall survival curves illustrating the prognostic value of TGFB1 gene expression, validated in TCGA HGSTOC cohorts.

(G)Kaplan–Meier overall survival curves illustrating the prognostic value of SIRT2 gene expression, validated in TCGA HGSTOC cohorts.

(H)A bar plot demonstrating the expression of HDAC1 in ovarian cancer cell lines (A2780, SKOV3, CAOV3, OVCAR4, NIHOVCAR3, ES2) and normal cell line (OELE).

(I) Relative Expression Levels of HDAC1 in ovarian cancer cell lines (CAOV3, OV8, NIHOVCAR3, A2780, SKOV3). The relative expression levels of HDAC1 in ovarian cancer cell lines were measured using qPCR. The expression levels were normalized to the housekeeping gene GADPH. Data are presented as mean ± SEM (n = 4). Significant differences between SKOV3 cell lines to other cell lines are indicated (****P < 0.0001).

HGSTOC, High grade serous tubo-ovarian carcinoma; HRP, Homologous recombination proficiency; HRD, Homologous recombination deficiency; qPCR, Quantitative Real-time PCR

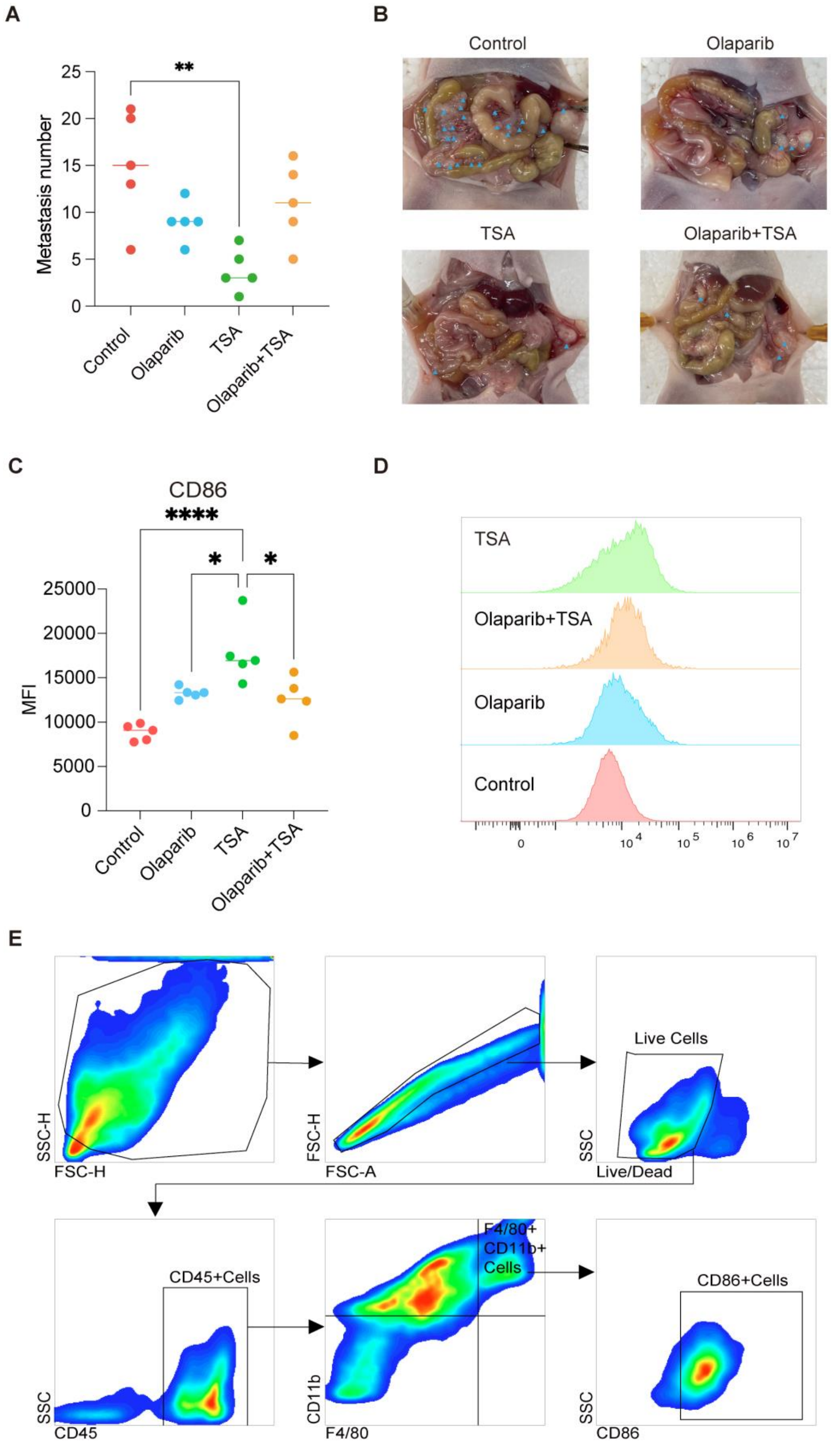

Figure S2: HDAC inhibitors demonstrate promising therapeutic effects for HRP tumors in intra-peritoneal xenograft tumor model

(A) A scatter plot illustrating the control of abdominal metastases in different treatment groups: HDAC inhibitor (TSA), PARPi (olaparib) monotherapy, PARPi

combined with HDAC inhibitor (olaparib+TSA), and the control group. Each point represents the number of metastatic foci in an individual mouse (n = 5 per group). The TSA group shows the greatest reduction in metastatic foci compared to the other treatment groups, indicating superior efficacy in controlling abdominal metastases. P value was calculated by one-way ANOVA analysis with Tukey's pairwise comparisons. **Adjusted P < 0.01

(B) Representative photos of intra-peritoneal xenograft tumor model showing decreased number of metastatic foci in TSA treated group than the control group. (n = 5 for each group)

(C) A scatter plot illustrating the MFI (Mean Fluorescence Intensity) values and (D)corresponding histogram overlays of the expression of CD86 in different treatment group.

(E) Flow cytometry gating strategy for identifying M1 cells. Cells were first gated by the forward and side scatter areas, and doublets were then excluded by gating with the forward scatter area and height. Live cells were gated by live/dead cell dye. Leukocytes were gated by CD45+ cells. Cells in the leukocyte gate were further gated based on F4/80+CD11b cells.

MFI, Mean Fluorescence Intensity ;TSA, Trichostatin A; PARPi, PARP inhibitor; HDACi, HDAC inhibitor

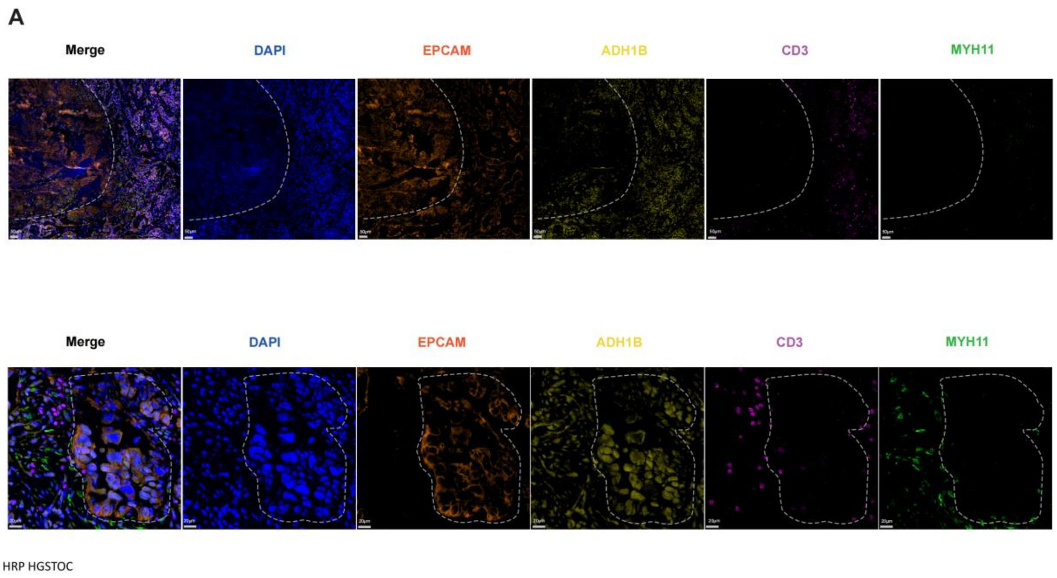

Figure S3. Immune-excluded ADH1B+ “indolent CAFs” in HRP group

(A)Multiplex immunohistochemistry showing a great abundance of indolent CAFs (ADH1B+, yellow) act as a barrier to exclude tumor epithelial cells (EPCAM+,

orange) from T cell (CD3+, mauve) infiltration with MYH11+ SMC inserted. Scale bar = 50 μM (upper), Scale bar = 20 μM (lower).

HGSTOC, High grade serous tubo-ovarian carcinoma; HRP, Homologous recombination proficiency; CAF, Cancer-associated fibroblasts; SMC, Smooth muscle cells

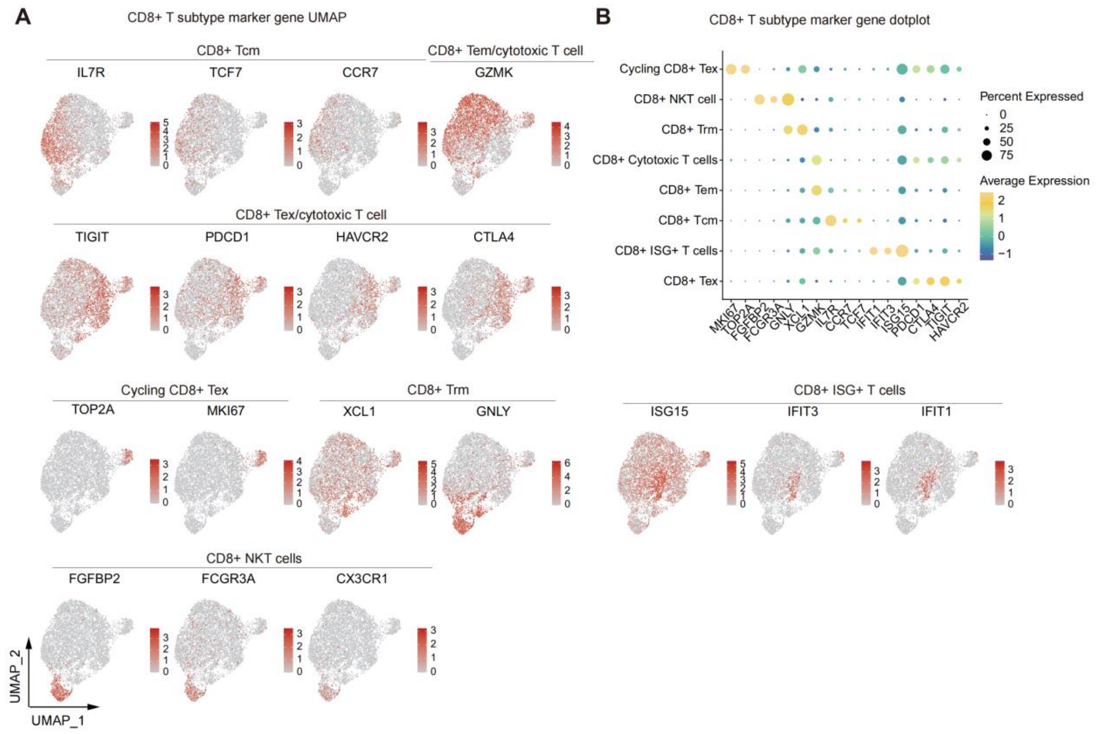

Figure S4. Distinct CD8+ T cell responses in HRD and HRP tumors: clonal expanded tumor-reactive T cells in HRD vs. bystander T cells in HRP

(A)UMAP plots demonstrating the classical markers of CD8+ T cell subtypes. Bar color represented the gene expression level (Red, higher expression; grey, lower expression).

(B)A dotplot showing the classical markers of CD8+ T cell subtypes. Dot size represented the percentage of marker gene expressed cells. Dot color represented the average expression level of marker genes (Yellow, higher expression; blue, lower expression).

HRP, Homologous recombination proficiency; HRD, Homologous recombination deficiency; UMAP, Uniform manifold approximation and projection

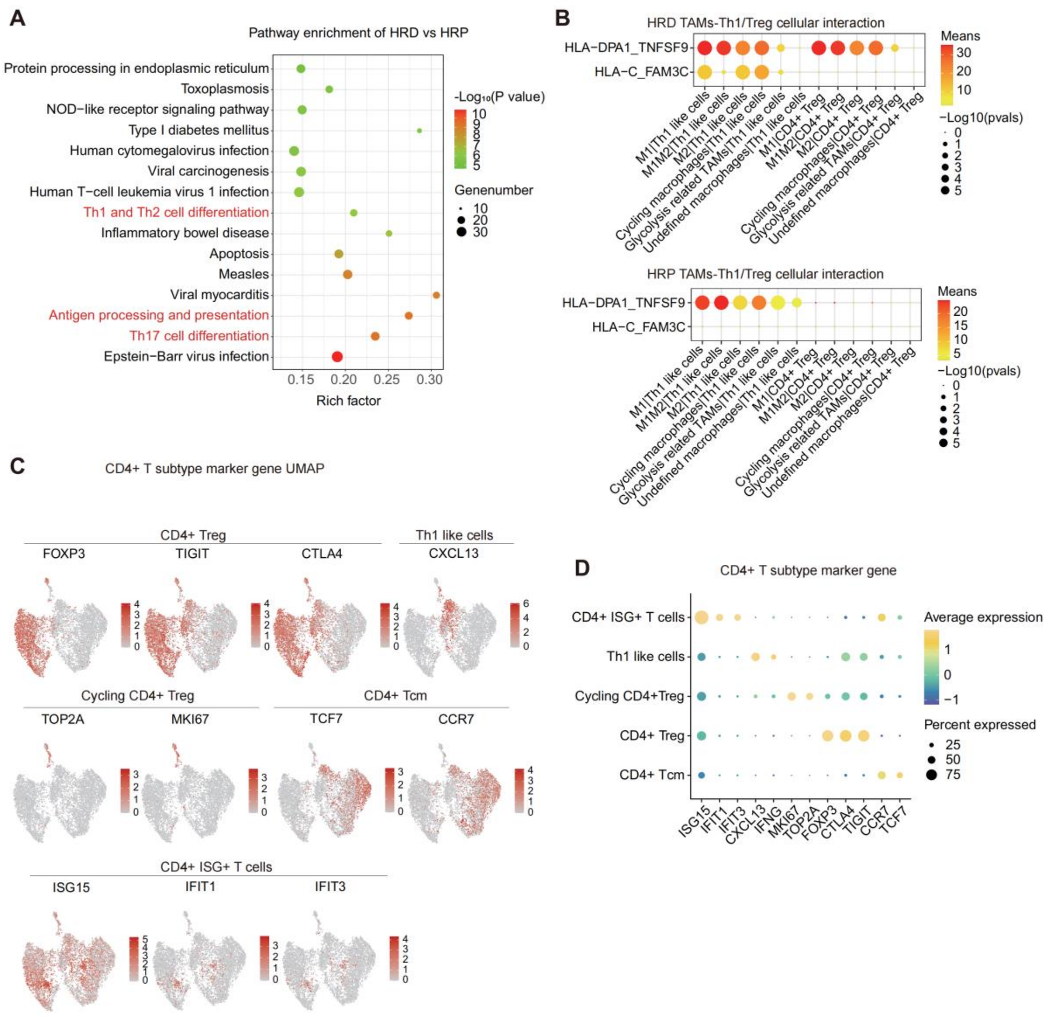

Figure S5. Distinct CD4+ T cell responses in HRD and HRP tumors: massive clonal expanded Treg in HRD vs. quiescent Tcm cells in HRP

(A)A dot plot demonstrating significant enriched pathways of CD4+T cells in HRD versus HRP tumors. P value was calculated by fisher exact test.

(B)A cellphonedb dot plot showing receptor-ligand interactions between TAMs to CD4+ T cells HRD and HRP. Dot size represented the p value of ligand-receptor interaction. Dot color represented the means of ligand-receptor interaction (Red, higher mean expression; yellow, lower mean expression).

(C)UMAP plots demonstrating the classical markers of CD4+ T cell subtypes. Bar color represented the gene expression level (Red, higher expression; grey, lower expression).

(D)A dotplot showing the classical markers of CD4+ T cell subtypes. Dot size represented the percentage of marker gene expressed cells. Dot color represented the average expression level of marker genes (Yellow, higher expression; blue, lower expression).

HRP, Homologous recombination proficiency; HRD, Homologous recombination deficiency; UMAP, Uniform manifold approximation and projection; TAM, Tumor-associated macrophages

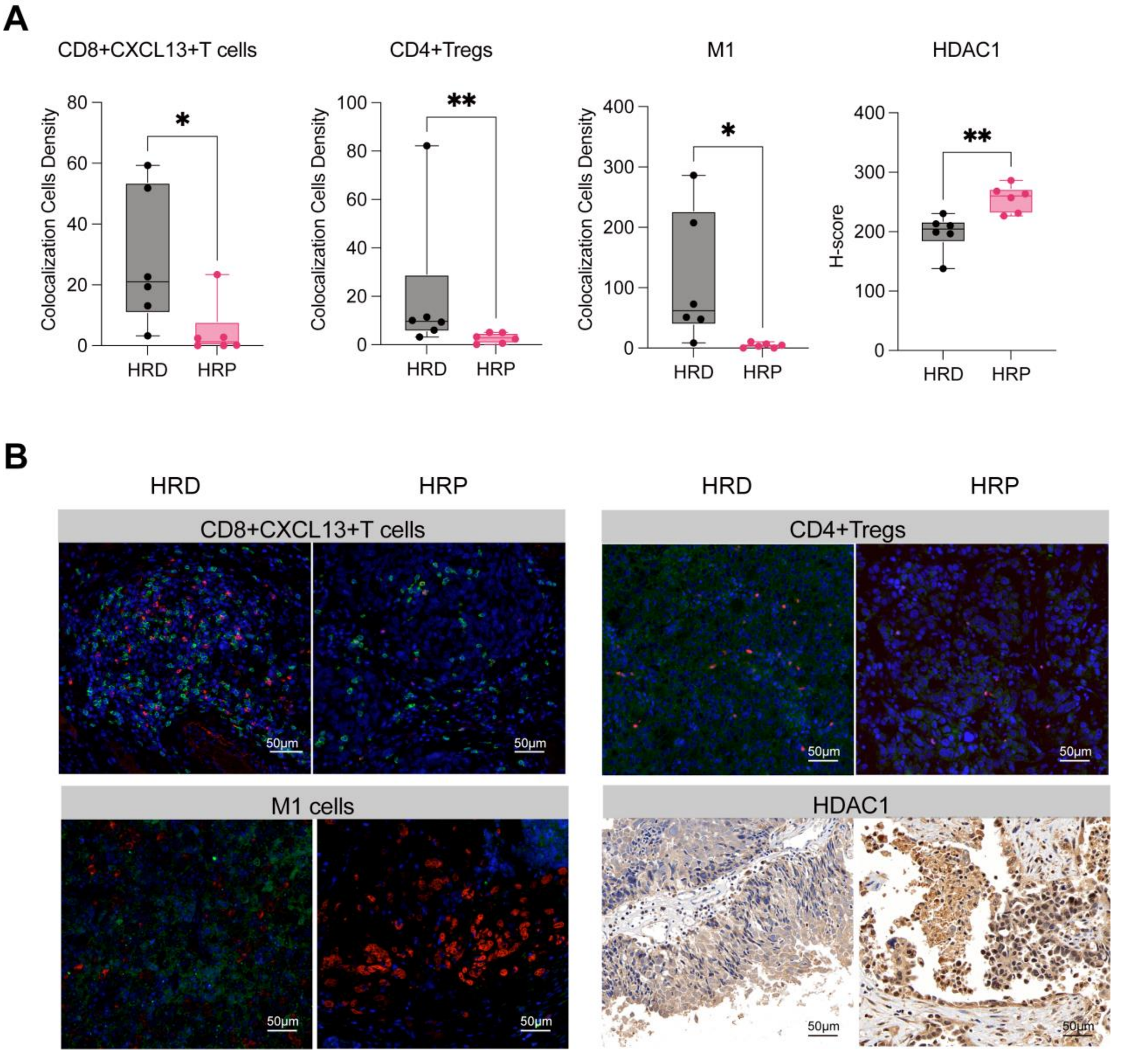

Figure S6. Multicolor immunofluorescence and immunohistochemistry validation of the infiltration level of CXCL13+ CD8+ T cells, CD4+ Treg cells and M1 cells as well as the expression level of HDAC1 in HRD and HRP samples

(A) A bar plot demonstrating the colocalization cells density of CXCL13+ CD8+ T cells, CD4+ Treg cells and M1 cells and H-score of HDAC1 expression in HRD and HRP samples(n=6 per group), P value was calculated by Mann-Whitney test (CD4+ Treg cells , CXCL13+ CD8+ T cells), unpaired t test (M1 cells, HDAC1).(*$P < 0.05$, **$P < 0.01$).

(B) Representative immunofluorescence images showing CXCL13+(red) CD8+(green) T cells, CD4+(green) Foxp3+(red) Treg cells, CD68+(red) CD86+(green) M1 cells and the expression levels of HDAC1(n=6 per group).Scale bar = 50 μM . Three fields of each slide were randomly picked.

HRP, Homologous recombination proficiency; HRD, Homologous recombination deficiency
